# Supplementary material for: Kinematic priming of action predictions
Source: Curr Biol. 2023 Jul 10;33(13):2717–2727.e6. doi: 10.1016/j.cub.2023.05.055 (PMC10357321; doi:10.1016/j.cub.2023.05.055)
Supplement: Document S1. Figures S1–S5 and Tables S1–S3 [file mmc1.pdf]

Current Biology, Volume *33*

Supplemental Information

Kinematic priming of action predictions

Eugenio Scaliti, Kiri Pullar, Giulia Borghini, Andrea Cavallo, Stefano Panzeri, and Cristina Becchio

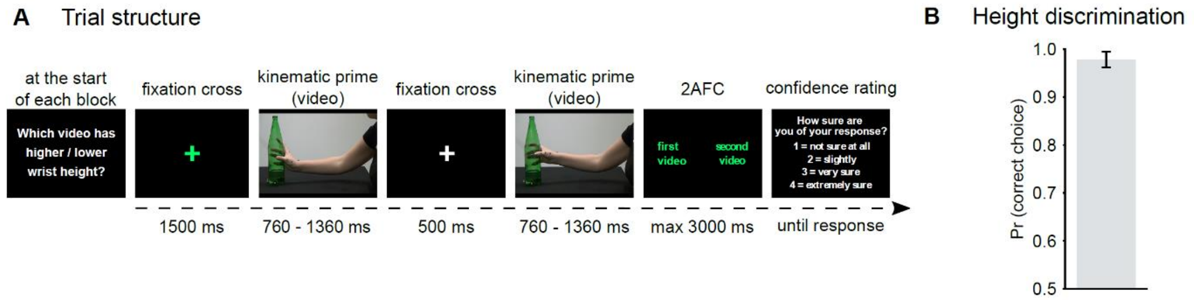

Figure S1. Wrist height discrimination task, Related to STAR Methods. Wrist height was the most informative kinematic variable and also the most readout variable. To verify that differences in wrist height were accessible to human perceivers, in a control study, we asked participants (N = 8) to discriminate wrist height in a 2AFC task. **A)** Trial structure. On each trial, participants observed two reach-to-grasp movements in two consecutive intervals. One interval contained a reach-to-pour, the other a reach-to-drink (in randomized order). Participants were asked to indicate the interval displaying the reach-to-grasp with higher peak wrist height. **B)** Wrist height discrimination performance quantified as the predicted probability of correct choice. The histogram shows the estimated marginal mean ± SE at the population-level estimated from a mixed model fit to single trial data.

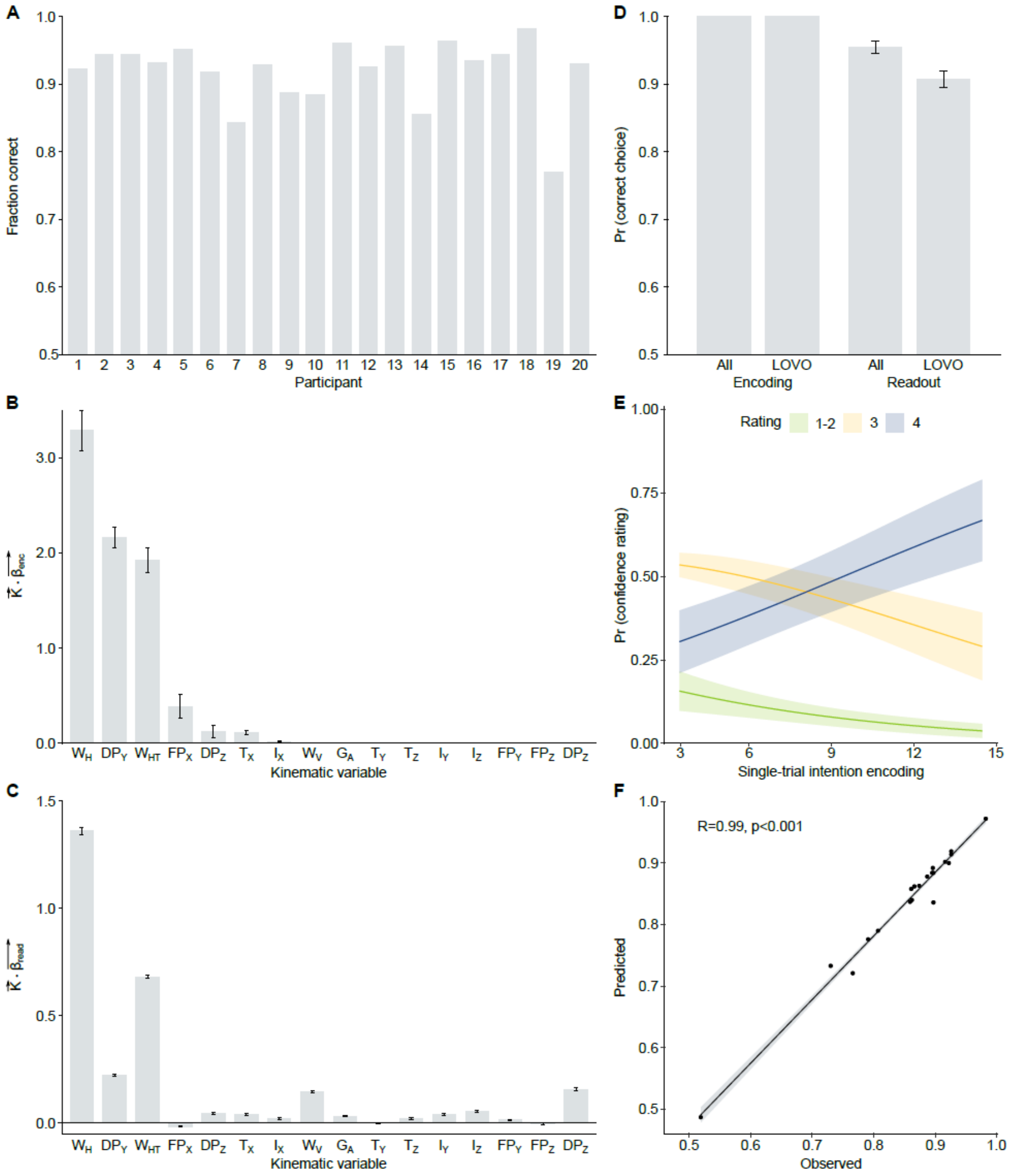

Figure S2. Kinematic intersection framework, Related to Figure 2. Here we summarize results of additional analyses to validate the modelling of intention encoding and intention readout using the kinematic intersection framework. **A)** Performance of the individual readout models trained on each participant. **B)** Contribution of individual kinematic variables to encoding computed as the scalar product between the encoding vector and the single-trial kinematic vector. Histograms represent mean ± SEM across kinematic primes. **C)** Contribution of individual kinematic variables to readout computed as the scalar product between the readout vector and the single-trial kinematic vector. Histograms represent mean ± SEM across trials and participants. **D)** Encoding (left) and readout (right) performance of models refit on entire data set after selection of λ parameter (ALL, as reported in main text) versus encoding/readout performance obtained using nested leave-one-video-out cross validation (LOVO). **E)** Probability of confidence rating in the intention discrimination task as a function of single-trial intention encoding. **F)** Pearson correlation between the observed and predicted (by the readout models) intention discrimination accuracies of individual perceivers. In Panels **E)**–**F)**, the line and shaded region correspond to estimated marginal mean ± SE estimated from the cumulative link mixed model and the linear model fit, respectively.

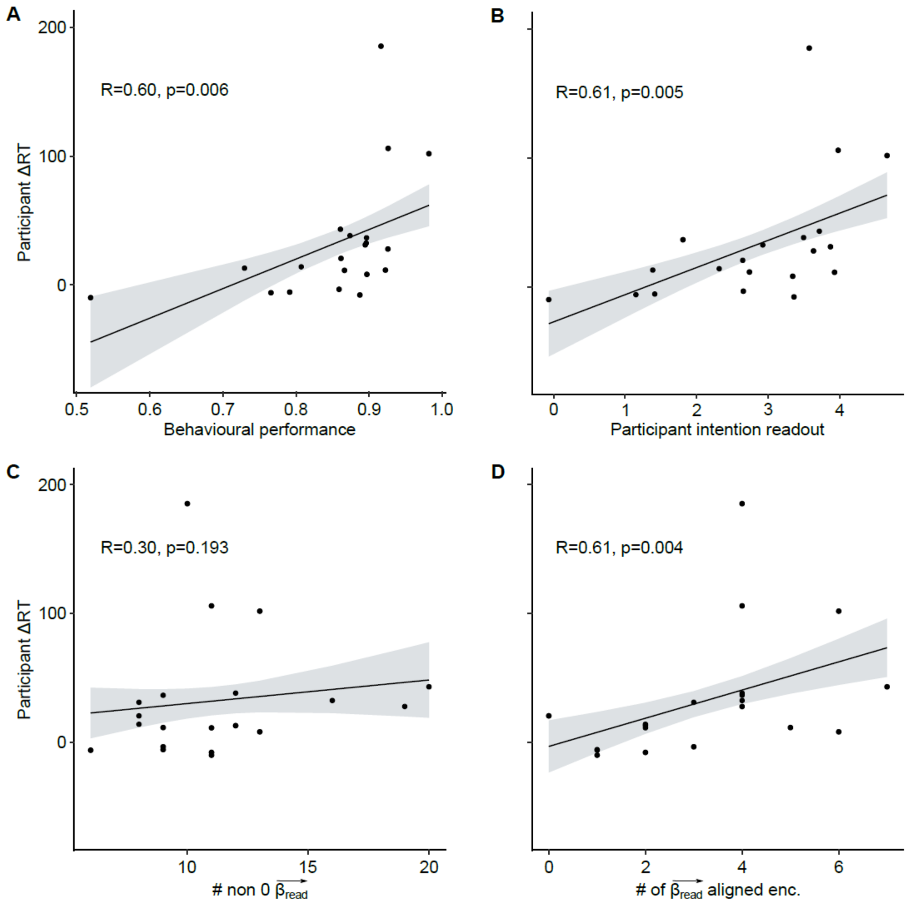

Figure S3. Intention readout predicts differences in kinematic priming across perceivers, Related to Figure 3. Here we summarize the results of several analyses that further validate the power of individual readout models trained on data from the intention discrimination task to predict RTs of the same perceiver in the primed action categorization task. **A)** Spearman correlation between individual intention discrimination performance and individual kinematic priming effect (RTs incongruent – RTs congruent). **B)** Spearman correlation between participant intention readout and individual kinematic priming effect. **C)** Spearman correlation between the number of non-zero readout coefficients and individual kinematic priming effect. **D)** Spearman correlation between the number of non-zero readout coefficients assigned to informative features and correctly aligned with encoding and individual kinematic priming effect. In all panels, the lines and shaded regions correspond to estimated marginal means ± SE estimated from linear model fit.

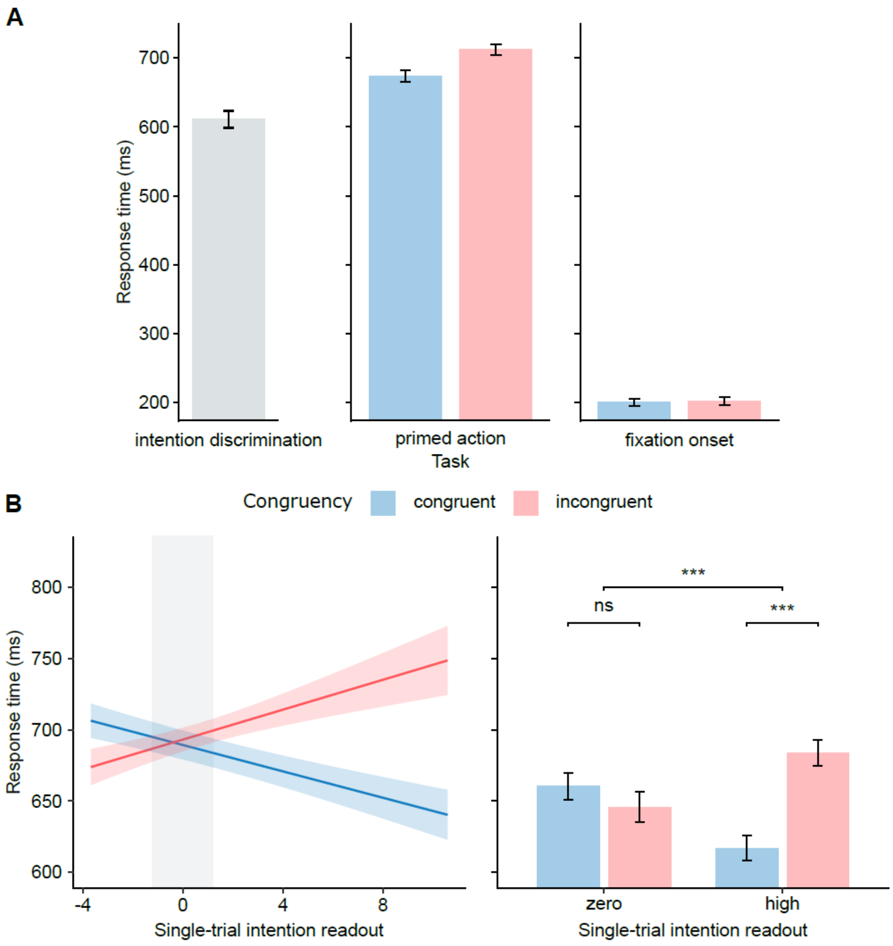

Figure S4. Additional analyses on response latencies, Related to Figures 3 and 4. Here we summarize the results of various analyses comparing response latencies across tasks and conditions. **A)** Left: RTs in the intention discrimination task. Middle: RTs in the primed action categorization task, separately for congruent and incongruent trials. Right: onset of initial fixation in the primed action categorization task, separately for congruent and incongruent trials. Initial fixations landed on the region predicted to contain task-relevant information approximately 200 ms after the probe was displayed. Because the time to discriminate kinematic primes in the intention discrimination task exceeded 600 ms, this observation refutes the idea that participants performed the primed action categorization task by first explicitly identifying the kinematic prime intention and then using this information to categorize the probe image intention. **B)** Left: RTs of the primed action categorization task plotted by single-trial intention readout and congruency. These data are the same as those in Fig 3B but are replotted here with the addition of the region (grey area) of single-trial intention readout values selected as zero-readout region for the selection of zero readout video primes. Right: RTs for the primed action categorization task plotted by congruency, separately for zero and high readout information. In comparison to zero-readout trials, RTs were faster on congruent trials and slower on incongruent trials in high-readout trials. This indicates that intention information read out in kinematic primes facilitated the processing of congruent probes and hindered the processing of incongruent probes.

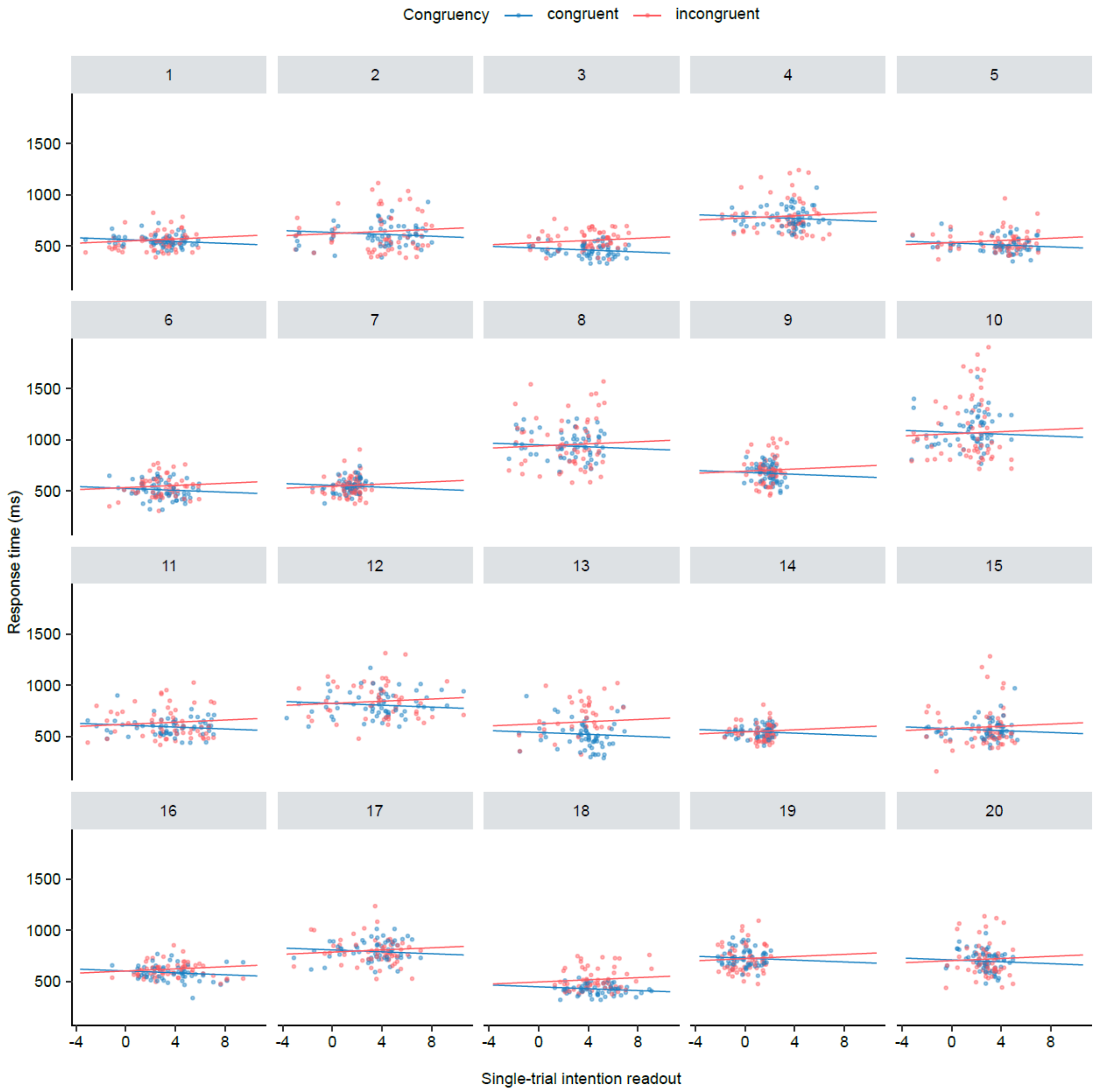

Figure S5. RT data for the primed action categorization task, Related to Figure 3. The figure displays the RT data for the primed action categorization task and demonstrates the consistency of the single-trial intention readout priming effect across individual perceivers (identified by grey boxes). Each panel depicts RTs by single-trial intention readout and congruency for each perceiver, with individual trials represented as data points. The red and blue lines plot the conditional marginal means estimated from the mixed model fit to single trial data for congruent and incongruent trials, respectively. The trend reported at the population level in Figure 3B is highly consistent across individual perceivers.

RTs: dependence on prime and probe (Fig. 1D)

	χ^2	df	p
**Probe**	**4.534**	**1**	**0.033**
Prime	1.493	1	0.222
**Probe:Prime**	**64.896**	**1**	**<0.001**

Intention discrimination accuracy (Fig. 1E)

	χ^2	df	p
**Prime**	**10.693**	**1**	**0.001**

Confidence ratings: dependence on single-trial intention encoding and readout (Fig. S2E and 2F)

	χ^2	df	p
**Encoding**	**4.839**	**1**	**0.0278**

	χ^2	df	p
**Readout**	**37.022**	**1**	**<0.001**

RTs: dependence on congruency and single-trial intention encoding and readout (Fig. 3A and 3B)

	χ^2	df	p
Encoding	0.060	1	0.807
Congr	3.441	1	0.064
Probe	0.333	1	0.564
Encoding:Congr	0.367	1	0.545
**Encoding:Probe**	**5.772**	**1**	**0.016**
Congr:Probe	0.336	1	0.562
Encoding:Congr:Probe	0.046	1	0.831

	χ^2	df	p
Readout	0.037	1	0.847
Congr	0.062	1	0.803
**Probe**	**4.405**	**1**	**0.036**
**Readout:Congr**	**12.749**	**1**	**<0.001**
Readout:Probe	0.526	1	0.468
Congr:Probe	0.877	1	0.349
Readout:Congr:Probe	0.109	1	0.741

RT Priming effect: comparison between low and high single-trial intention encoding and readout (Fig. 3C and 3D)

	χ^2	df	p
EncLev	0.188	1	0.664
**Congr**	**59.279**	**1**	**<0.001**
**Probe**	**4.386**	**1**	**0.036**
EncLev:Congr	0.439	1	0.508
**EncLev:Probe**	**4.328**	**1**	**0.037**
Congr:Probe	1.354	1	0.245
EncLev:Congr:Probe	0.576	1	0.448

	χ^2	df	p
ReadLev	0.004	1	0.950
**Congr**	**59.221**	**1**	**<0.001**
**Probe**	**24.491**	**1**	**<0.001**
**ReadLev:Congr**	**36.475**	**1**	**<0.001**
ReadLev:Probe	1.018	1	0.313
Congr:Probe	1.344	1	0.246
ReadLev:Congr:Probe	0.070	1	0.791

Fixation in quadrant relevant for displayed probe: dependence on prime and probe (logit scale; Fig. 4B)

	χ^2	df	p
Probe	0.445	1	0.505
Prime	0.569	1	0.451
**Probe:Prime**	**60.961**	**1**	**<0.001**

Fixation in quadrant relevant for not displayed probe: dependence on prime and probe (logit scale; Fig. 4C)

	χ^2	df	p
Probe	1.123	1	0.289
Prime	1.347	1	0.246
**Probe:Prime**	**53.190**	**1**	**<0.001**

Initial fixations: dependence on single-trial intention encoding and readout (logit scale; Fig. 5A and 5B)

	χ^2	df	p
Encoding	0.832	1	0.362
Congr	0.812	1	0.368
Probe	1.433	1	0.231
Encoding:Congr	2.379	1	0.123
Encoding:Probe	1.018	1	0.313
Congr:Probe	2.944	1	0.086
**Encoding:Congr:Probe**	**4.448**	**1**	**0.035**

	χ^2	df	p
Readout	3.669	1	0.055
Congr	2.709	1	0.100
Probe	0.100	1	0.751
**Readout:Congr**	**11.445**	**1**	**<0.001**
Readout:Probe	0.004	1	0.947
Congr:Probe	1.545	1	0.214
Readout:Congr:Probe	3.171	1	0.075

Initial fixations priming effect: comparison between low and high intention encoding and readout (logit scale; Fig. 5C and 5D)

	χ^2	df	p
EncLev	1.010	1	0.315
**Congr**	**58.021**	**1**	**<0.001**
Probe	0.514	1	0.474
EncLev:Congr	2.448	1	0.118
EncLev:Probe	0.945	1	0.331
Congr:Probe	0.824	1	0.364
EncLev:Congr:Probe	1.052	1	0.305

	χ^2	df	p
ReadLev	0.394	1	0.530
**Congr**	**60.179**	**1**	**<0.001**
Probe	0.213	1	0.644
**ReadLev:Congr**	**13.031**	**1**	**<0.001**
ReadLev:Probe	0.604	1	0.437
Congr:Probe	0.330	1	0.566
ReadLev:Congr:Probe	0.061	1	0.805

RT Priming effect: comparison between zero and high single-trial intention readout (Fig. S4B)

	χ^2	df	p
ZeroRead	0.068	1	0.794
**Congr**	**13.103**	**1**	**<0.001**
**ZeroRead:Congr**	**27.620**	**1**	**<0.001**

Pooled participants: RTs dependence on congruency and single-trial intention readout (not plotted in Figures)

	χ^2	df	p
Readout	0.143	1	0.706

Pupil dilation: RTs dependence on congruency and single-trial intention readout (not plotted in Figures)

	χ^2	df	p
Readout	1.012	1	0.314

**Congr**	**8.119**	**1**	**0.004**	Congr	0.085	1	0.770
Probe	0.002	1	0.969	**Pupil**	**14.799**	**1**	**<0.001**
Readout:Congr	0.734	1	0.392	**Reaout:Congr**	**26.107**	**1**	**<0.001**
**Readout:Probe**	**6.675**	**1**	**0.010**	Readout:Pupil	0.134	1	0.714
Congr:Probe	0.102	1	0.750	Congr:Pupil	2.539	1	0.111
Readout:Congr:Probe	0.174	1	0.677	Readout:Congr:Pupil	0.004	1	0.951

Table S1. Summary of likelihood ratio tests for significance of main effects and product terms in mixed effects statistics, Related to Figures 1-5.

RTs: dependence on prime and probe (Fig. 1D)

RT~Probe*Prime+(1+Probe|participant)+(1|prime)

	est	SE	z	p
**(Intercept)**	**692.843**	**7.607**	**91.077**	**<0.001**
**Probe1**	**-12.593**	**4.558**	**-2.763**	**0.006**
Prime1	-4.483	3.617	-1.240	0.215
**Probe1:Prime1**	**-18.984**	**2.331**	**-8.144**	**<0.001**

Intention discrimination accuracy (Fig.1E)

Accuracy~Prime+(1+Prime|participant)+(1|prime)

	est	SE	z	p
**(Intercept)**	**2.804**	**0.284**	**9.870**	**<0.001**
**Prime1**	**-0.899**	**0.261**	**-3.440**	**<0.001**

Confidence ratings: dependence on single-trial intention encoding and readout (Fig. S2E and 2F)

ConfRating~Encoding+(1+Encoding|participant)+(1|prime)

	est	SE	z	p
**Encoding**	**0.138**	**0.062**	**2.235**	**0.025**
Threshold coeff 1-2|3	-1.191	0.593	-2.008	
Threshold coeff 3|4	1.298	0.593	2.188	

ConfRating~Readout+(1+Readout|participant)+(Readout|prime)

	est	SE	z	p
**Readout**	**0.700**	**0.076**	**9.221**	**<0.001**
Threshold coeff 1-2|3	-0.414	0.375	-1.104	
Threshold coeff 3|4	2.372	0.378	6.278	

RTs: dependence on congruency and single-trial intention encoding and readout (Fig. 3A and 3B)

RT~Encoding*Congr*Probe+(1+Probe|participant)+(1|prime)

	est	SE	z	p
**(Intercept)**	**689.894**	**9.443**	**73.057**	**<0.001**
Encoding	0.347	1.344	0.258	0.797
**Congr1**	**-13.424**	**5.992**	**-2.240**	**0.025**
Probe1	5.603	6.253	0.896	0.370
Encoding:Congr1	-0.567	0.744	-0.763	0.445
**Encoding:Probe1**	**-2.257**	**0.756**	**-2.984**	**0.003**
Congr1:Probe1	-7.021	7.960	-0.882	0.378
Encoding:Congr1:Probe1	0.303	1.007	0.301	0.763

RT~Readout*Congr*Probe+(1+Congr|participant)+(1|prime)

	est	SE	z	p
**(Intercept)**	**691.206**	**7.533**	**91.761**	**<0.001**
Readout	0.308	1.495	0.206	0.837
Congr1	-1.884	5.280	-0.357	0.721
**Probe1**	**-9.795**	**3.508**	**-2.792**	**0.005**
**Readout:Congr1**	**-4.942**	**1.203**	**-4.108**	**<0.001**
Readout:Probe1	-0.913	1.039	-0.879	0.379
Congr1:Probe1	-5.295	4.356	-1.215	0.224
Readout:Congr1:Probe1	0.461	1.196	0.386	0.700

RT Priming effect: comparison between low and high single-trial intention encoding and readout (Fig. 3C and 3D)

RT~EncLev*Congr*Probe+(1+Probe|participant)+(1|prime)

	est	SE	z	p
**(Intercept)**	**692.446**	**7.288**	**95.015**	**<0.001**
EncLev1	1.585	3.081	0.514	0.607
**Congr1**	**-18.272**	**2.301**	**-7.942**	**<0.001**
**Probe1**	**-12.358**	**3.972**	**-3.111**	**0.002**
EncLev1:Congr1	1.599	2.251	0.710	0.477
**EncLev1:Probe1**	**5.034**	**2.254**	**2.233**	**0.026**
Congr1:Probe1	-4.268	3.206	-1.331	0.183
EncLev1:Congr1:Probe1	-2.780	3.370	-0.825	0.409

RT~ReadLev*Congr*Probe+(1+Congr|participant)+(1|prime)

	est	SE	z	p
**(Intercept)**	**691.182**	**5.768**	**119.822**	**<0.001**
ReadLev1	0.213	2.836	0.075	0.940
**Congr1**	**-18.606**	**2.345**	**-7.933**	**<0.001**
**Probe1**	**-12.219**	**2.232**	**-5.474**	**<0.001**
**ReadLev1:Congr1**	**14.922**	**2.217**	**6.730**	**<0.001**
ReadLev1:Probe1	2.485	2.306	1.077	0.281
Congr1:Probe1	-4.242	3.322	-1.277	0.202
ReadLev1:Congr1:Probe1	-0.716	2.450	-0.292	0.770

Fixation in quadrant relevant for displayed probe: dependence on prime and probe (Fig. 4B)

Fixation~Probe*Prime+(1+Probe|participant)+(1|prime)

	est	SE	z	p
**(Intercept)**	**0.644**	**0.211**	**3.050**	**0.002**
Probe1	-0.119	0.177	-0.671	0.502
Prime1	-0.042	0.055	-0.758	0.448
**Probe1:Prime1**	**0.340**	**0.043**	**7.854**	**<0.001**

Fixation in quadrant relevant for not displayed probe: dependence on prime and probe (Fig. 4C)

Fixation~Probe*Prime+(1+Probe|participant)+(1|prime)

	est	SE	z	p
**(Intercept)**	**-2.598**	**0.145**	**-17.891**	**<0.001**
Probe1	-0.239	0.222	-1.079	0.281
Prime1	-0.092	0.078	-1.182	0.237
**Probe1:Prime1**	**-0.472**	**0.062**	**-7.586**	**<0.001**

Initial fixations: dependence on single-trial intention encoding and readout (logit scale; Fig. 5A and 5B)

Fixation~Encoding*Congr*Probe+(1+Probe|participant)+(1|prime)

	est	SE	z	p
(Intercept)	0.516	0.267	1.932	0.053
Encoding	0.019	0.021	0.917	0.359
Congr1	0.127	0.140	0.906	0.365
Probe1	-0.268	0.222	-1.207	0.227
Encoding:Congr1	0.026	0.017	1.550	0.121
Encoding:Probe1	0.017	0.017	1.013	0.311
Congr1:Probe1	0.299	0.172	1.732	0.083
**Encoding:Congr1:Probe1**	**-0.044**	**0.021**	**-2.138**	**0.032**

Fixation~Readout*Congr*Probe+(1+Probe|participant)+(1|prime)

	est	SE	z	p
**(Intercept)**	**0.471**	**0.227**	**2.072**	**0.038**
Readout	0.052	0.027	1.926	0.054
Congr1	0.132	0.080	1.657	0.098
Probe1	-0.059	0.187	-0.317	0.751
**Readout:Congr1**	**0.075**	**0.022**	**3.387**	**<0.001**
Readout:Probe1	-0.002	0.024	-0.066	0.947
Congr1:Probe1	0.112	0.090	1.249	0.212
Readout:Congr1:Probe1	-0.042	0.024	-1.789	0.074

Initial fixations priming effect: comparison between low and high intention encoding and readout (logit scale; Fig. 5C and 5D)

Fixation~EncLev*Congr*Probe+(1+Probe|participant)+(1|prime)

	est	SE	z	p
**(Intercept)**	**0.652**	**0.211**	**3.088**	**0.002**
EncLev1	-0.055	0.054	-1.010	0.312
**Congr1**	**0.335**	**0.044**	**7.669**	**<0.001**
Probe1	-0.128	0.118	-0.721	0.471
EncLev1:Congr1	-0.068	0.043	-1.571	0.116
EncLev1:Probe1	-0.042	0.043	-0.976	0.329
Congr1:Probe1	-0.050	0.055	-0.913	0.361
EncLev1:Congr1:Probe1	0.056	0.054	1.032	0.302

Fixation~ReadLev*Congr*Probe+(1+Probe|participant)+(1|prime)

	est	SE	z	p
**(Intercept)**	**0.643**	**0.214**	**3.008**	**0.003**
ReadLev1	-0.036	0.056	-0.631	0.528
**Congr1**	**0.352**	**0.045**	**7.815**	**<0.001**
Probe1	-0.083	0.178	-0.464	0.643
**ReadLev1:Congr1**	**-0.165**	**0.046**	**-3.619**	**<0.001**
ReadLev1:Probe1	-0.041	0.053	-0.781	0.435
Congr1:Probe1	-0.032	0.056	-0.576	0.564
ReadLev1:Congr1:Probe1	-0.012	0.048	-0.248	0.804

Wrist height discrimination accuracy (Fig. S1B)

Accuracy~1+(1|participant)

	est	SE	z	p
**(Intercept)**	**3.812**	**0.776**	**4.909**	**<0.001**

RT Priming effect: comparison between zero and high single-trial intention readout (Fig. S4B)

RT~ZeroRead*Congr*Probe+(1+Probe|participant)+(1|prime)

	est	SE	z	p
**(Intercept)**	**651.604**	**6.958**	**93.644**	**<0.001**
ZeroRead1	1.349	4.428	0.305	0.761
**Congr1**	**-13.064**	**3.286**	**-3.976**	**<0.001**
**ZeroRead1:Congr1**	**20.427**	**3.490**	**5.853**	**<0.001**

Pooled participants: RTs dependence on congruency and single-trial intention readout (not plotted in Figures)

RT~Readout*Congr*Probe+(1+Probe|participant)+(1|prime)

	est	SE	z	p
**(Intercept)**	**694.143**	**6.072**	**114.326**	**<0.001**
Readout	-1.238	2.587	-0.478	0.632
**Congr1**	**-18.352**	**4.153**	**-4.419**	**<0.001**
Probe1	-0.322	4.127	-0.078	0.938
Readout:Congr1	-2.247	1.852	-1.214	0.225
**Readout:Probe1**	**-6.853**	**1.790**	**-3.828**	**<0.001**
Congr1:Probe1	-2.550	4.532	-0.563	0.574
Readout:Congr1:Probe1	-1.336	2.085	-0.641	0.522

Pupil dilation: RTs dependence on congruency and single-trial intention readout (not plotted in Figures)

RT~Readout*Congr*Pupil+(1|participant)+(1+Probe|prime)

	est	SE	z	p
**(Intercept)**	**668.453**	**7.264**	**92.017**	**<0.001**
Readout	1.406	1.319	1.066	0.286
Congr1	-1.611	4.143	-0.389	0.697
**Pupil**	**14.058**	**3.206**	**4.385**	**<0.001**
**Readout:Congr1**	**-6.402**	**1.150**	**-5.565**	**<0.001**
Readout:Pupil	-0.362	0.902	-0.401	0.688
Congr1:Pupil	5.656	3.283	1.722	0.085
Readout:Congr1:Pupil	0.061	0.925	0.066	0.948

Table S2. Summary of model formula and fixed coefficients for mixed effects statistics, Related to Figures 1-5.

RTs: effects of prime (Fig. 1D)

probe	est	SE	z	p
**drinking**	**-46.935**	**8.630**	**-5.439**	**<0.001**
**pouring**	**29.002**	**8.581**	**3.380**	**<0.001**

RTs: difference in priming effect (Fig. 1D)

	est	SE	z	p
**drinking -pouring**	**-75.937**	**9.324**	**-8.144**	**<0.001**

Intention discrimination (prob. scale; Fig. 1E)

	est	SE	z	p
**vs. chance (0.5)**	**0.943**	**0.015**	**28.958**	**<0.001**

RTs: effects of single-trial intention encoding and readout (Fig. 3A and 3B)

	Encoding trend	SE	z	p
congruent	-0.221	1.312	-0.168	1.000
incongruent	0.914	1.731	0.528	1.000

	Readout trend	SE	z	p
**congruent**	**-4.634**	**1.471**	**-3.151**	**0.003**
**incongruent**	**5.250**	**2.281**	**2.302**	**0.021**

RTs: effects of congruency (Fig. 3A and 3B)

	est	SE	z	p
incongruent - congruent	1.135	1.487	0.763	0.445

	est	SE	z	p
**incongruent - congruent**	**9.884**	**2.406**	**4.108**	**<0.001**

RT Priming effect: effects of low and high single-trial intention encoding and readout (Fig. 3C and 3D)

EncLev	est	SE	z	p
**low**	**33.345**	**6.446**	**5.173**	**<0.001**
**high**	**39.742**	**6.430**	**6.181**	**<0.001**

ReadLev	est	SE	z	p
low	7.369	6.476	1.138	0.255
**high**	**67.056**	**6.434**	**10.422**	**<0.001**

RT Priming effect: low vs. high single-trial intention encoding and readout (Fig. 3C and 3D)

	est	SE	z	p
low - high	-6.397	9.005	-0.710	0.477

	est	SE	z	p
**low - high**	**-59.686**	**8.869**	**-6.730**	**<0.001**

Fixation in quadrant relevant for displayed probe: effect of prime (prob. scale; Fig. 4B)

probe	est	SE	z	p
**drinking**	**0.138**	**0.035**	**4.002**	**<0.001**
**pouring**	**-0.164**	**0.034**	**-4.784**	**<0.001**

Fixation in quadrant relevant for displayed probe: difference in priming effect (prob. scale; Fig. 4B)

	est	SE	z	p
**drinking -pouring**	**0.303**	**0.044**	**6.827**	**<0.001**

Fixation in quadrant relevant for not displayed probe: effect of prime (prob. scale; Fig. 4C)

probe	est	SE	z	p
**drinking**	**-0.061**	**0.019**	**-3.281**	**0.002**
**pouring**	**0.061**	**0.021**	**2.872**	**0.004**

Fixation in quadrant relevant for not displayed probe: difference in priming effect (prob. scale; Fig. 4C)

	est	SE	z	p
**drinking -pouring**	**-0.122**	**0.024**	**-5.153**	**<0.001**

Initial fixation: effects of single-trial intention encoding and readout (prob. scale; Fig. 5A and 5B)

	Encoding trend	SE	z	p
congruent	0.008	0.004	1.934	0.106
incongruent	-0.002	0.007	-0.203	0.839

	Readout trend	SE	z	p
**congruent**	**0.025**	**0.007**	**3.822**	**<0.001**
incongruent	-0.006	0.010	-0.600	0.549

Initial fixations: effect of congruency (prob. scale; Fig. 5A and 5B)

	est	SE	z	p
incongruent - congruent	-0.010	0.008	-1.265	0.206

	est	SE	z	p
**incongruent - congruent**	**-0.031**	**0.011**	**-2.895**	**0.004**

Initial fixations: effects of low and high single-trial intention encoding and readout (prob. scale; Fig. 5C and 5D)

EncLev	est	SE	z	p
**low**	**-0.121**	**0.029**	**-4.138**	**<0.001**
**high**	**-0.175**	**0.031**	**-5.682**	**<0.001**

ReadLev	est	SE	z	p
**low**	**-0.084**	**0.030**	**-2.791**	**0.005**
**high**	**-0.227**	**0.030**	**-6.904**	**<0.001**

Initial fixations: low vs. high intention single trial intention encoding and readout (prob. scale; Fig. 5C and 5D)

	est	SE	z	p
low - high	0.054	0.041	1.331	0.183

	est	SE	z	p
**low - high**	**0.143**	**0.043**	**3.316**	**<0.001**

Wrist height discrimination (prob. scale; Fig. S1B)

	est	SE	z	p
**vs. chance (0.5)**	**0.978**	**0.0164**	**29.113**	**<.0001**

RT Priming effect: effects of zero and high single-trial intention readout (Fig. S4B)

	est	SE	z	p
zero	-14.726	11.759	-1.252	0.210
**high**	**66.982**	**6.749**	**9.925**	**<0.001**

RT readout information effect: effects of congruent and incongruent trials (Fig. S4B)

	est	SE	z	p
**congruent**	**-43.552**	**9.740**	**-4.471**	**<0.001**
**incongruent**	**38.156**	**12.625**	**3.022**	**0.005**

Pooled participants: RTs effects of single-trial intention readout (not plotted in Figures)

	Readout trend	SE	z	p
congruent	-3.485	2.664	-1.308	0.382
incongruent	1.010	3.625	0.279	0.781

Pupil dilation: RTs effects of single-trial intention readout (not plotted in Figures)

	Readout trend	SE	z	p
**congruent**	**-4.943**	**1.390**	**-3.555**	**<0.001**
**incongruent**	**7.882**	**2.034**	**3.876**	**<0.001**

Pooled participants: RTs effects of congruency (not plotted in Figures)

	est	SE	z	p
incongruent - congruent	4.495	3.704	1.214	0.225

Pupil dilation: RTs effects of congruency (not plotted in Figures)

	est	SE	z	p
**incongruent - congruent**	**12.825**	**2.292**	**5.596**	**<0.001**

Table S3. Summary of post-hoc tests of main effects/trends and interactions for mixed model analyses, Related to Figures 1-5.
