# Supplementary material for: Kinematic priming of action predictions
Source: Curr Biol. 2023 Jul 10;33(13):2717–2727.e6. doi: 10.1016/j.cub.2023.05.055 (PMC10357321; doi:10.1016/j.cub.2023.05.055)
Supplement: Document S2. Article plus supplemental information [file mmc4.pdf]

Current Biology

Article

Kinematic priming of action predictions

Highlights

- Novel kinematic priming reveals rapid, implicit access to intention information
- Intention information in movement kinematic primes action prediction
- Single-trial intention readout predicts the amount of kinematic priming

Authors

Eugenio Scaliti, Kiri Pullar, Giulia Borghini, Andrea Cavallo, Stefano Panzeri, Cristina Becchio

Correspondence

s.panzeri@uke.de (S.P.),
c.becchio@uke.de (C.B.)

In brief

To navigate the social environment, we often need to predict the goals and intentions of others. Scaliti et al. demonstrate that movement kinematics provides human perceivers with rapid, implicit access to intention information.

Scaliti et al., 2023, Current Biology *33*, 2717–2727
July 10, 2023 © 2023 The Authors. Published by Elsevier Inc.
https://doi.org/10.1016/j.cub.2023.05.055

CellPress

Current Biology

CellPress
OPEN ACCESS

Article

Kinematic priming of action predictions

Eugenio Scaliti,[1,2,5] Kiri Pullar,[1,5] Giulia Borghini,[1] Andrea Cavallo,[1,3] Stefano Panzeri,[1,4,*] and Cristina Becchio[1,2,6,*]
[1]Center for Human Technologies, Fondazione Istituto Italiano di Tecnologia, Via Enrico Melen, 83, 16152 Genova, Italy
[2]Department of Neurology, University Medical Center Hamburg-Eppendorf (UKE), Martinistrasse 52, 20246 Hamburg, Germany
[3]Department of Psychology, Università degli Studi di Torino, Via Giuseppe Verdi, 10, 10124 Torino, Italy
[4]Department of Excellence for Neural Information Processing, Center for Molecular Neurobiology (ZMNH), University Medical Center Hamburg-Eppendorf (UKE), Falkenried 94, 20251 Hamburg, Germany
[5]These authors contributed equally
[6]Lead contact
*Correspondence: s.panzeri@uke.de (S.P.), c.becchio@uke.de (C.B.)
https://doi.org/10.1016/j.cub.2023.05.055

SUMMARY

The ability to anticipate what others will do next is crucial for navigating social, interactive environments. Here, we develop an experimental and analytical framework to measure the implicit readout of prospective intention information from movement kinematics. Using a primed action categorization task, we first demonstrate implicit access to intention information by establishing a novel form of priming, which we term kinematic priming: subtle differences in movement kinematics prime action prediction. Next, using data collected from the same participants in a forced-choice intention discrimination task 1 h later, we quantify single-trial intention readout—the amount of intention information read by individual perceivers in individual kinematic primes—and assess whether it can be used to predict the amount of kinematic priming. We demonstrate that the amount of kinematic priming, as indexed by both response times (RTs) and initial fixations to a given probe, is directly proportional to the amount of intention information read by the individual perceiver at the single-trial level. These results demonstrate that human perceivers have rapid, implicit access to intention information encoded in movement kinematics and highlight the potential of our approach to reveal the computations that permit the readout of this information with single-subject, single-trial resolution.

INTRODUCTION

Motor control anticipates future states. In tasks where an object is reached, grasped, lifted, and manipulated, subtle changes in reaching behavior anticipate the actor's intention in grasping the object.[1] This raises the possibility that others' goals and intentions can be inferred from movement kinematics.[1–4] But do human perceivers have access to this information? Do they use prospective information (i.e., information about the behavior to follow) encoded in movement kinematics to predict the actions of others?

Research addressing these questions has focused on paradigms using the forced-choice format.[5] In a typical experiment, a video of a reaching movement is played, and participants are asked to decide on the intention of the observed movement, e.g., whether the observed reach is guided by the intent to pour or drink.[6–9] Forced decisions of this type are a sensitive measure of intention readout. They allow researchers to determine how well perceivers can discriminate between actions performed with different intentions.[6,8] Combined with computational methods, they enable assessment of how intention information encoded in movement kinematics (single-trial kinematic encoding) is read out by individual human perceivers (single-trial kinematic readout) at the single-trial level.[10–12]

Studies using this approach show that naive participants are able to read some, but not all, of the prospective information encoded in movement kinematics.[11] However, this does not mean that human perceivers normally represent (or use) this information. By design, in a two-alternative forced-choice task, participants are forced to make a choice between alternatives that have to differ. Their choice can be based on separable representations of the two action intentions or simply on differences in movement kinematics or even differences in a specific kinematic variable. Thus, it remains unclear whether human perceivers represent action intentions (even without explicit instruction) or if they simply rely on variations in movement kinematics to distinguish between different alternatives.[13]

Priming effects have long been used as tools for probing internal representations.[14–17] Here, we introduce a novel priming technique—kinematic priming—to test whether perceivers build a representation of others' intentions from observing their movements. Priming occurs when the judgment of a target stimulus (probe) is facilitated by the prior presentation of a prime stimulus.[14] We reasoned that if mere exposure to movement kinematics is sufficient to activate an internal representation of the agent's intention in reaching the object, then we should observe kinematic priming: exposure to a kinematic prime encoding a given intention should facilitate the subsequent processing of an action performed with the same intention.

This is an open access article under the CC BY license (http://creativecommons.org/licenses/by/4.0/).

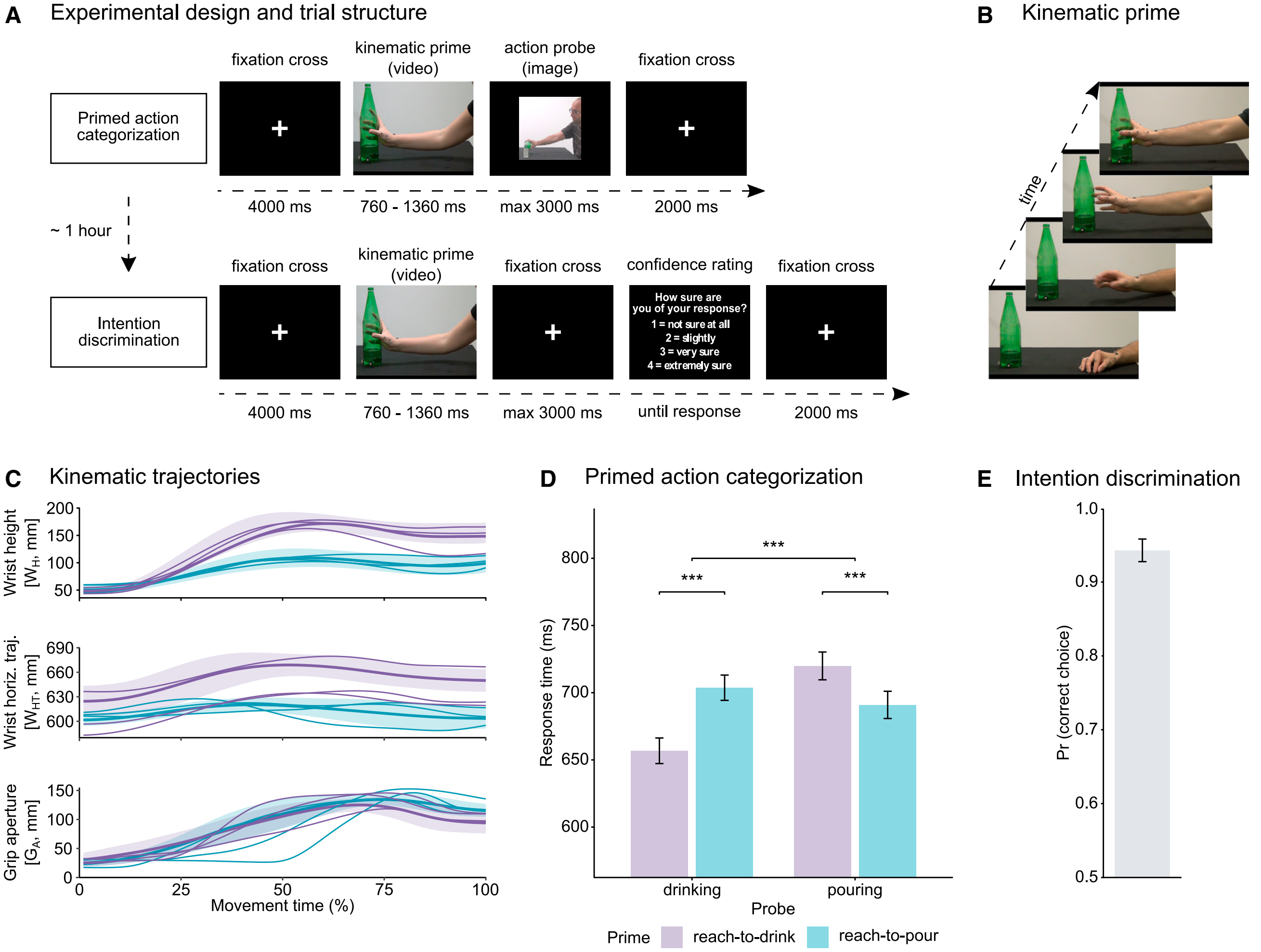

Figure 1. Experimental design and results of kinematic priming and intention discrimination

(A) Experimental design and trial structure. Participants performed a primed action categorization task and, 1 h later, a forced-choice intention discrimination task.

(B) Kinematic primes consisted of videos of reach-to-drink and reach-to-pour movements.

(C) Time course of wrist height (W_H), wrist horizontal trajectory (W_{HT}), and grip aperture (G_A) for reach-to-drink and reach-to-pour movements. Thin curves show representative individual trajectories; thick curves with shaded areas show the mean ± SD across kinematic prime stimuli.

(D) Response times (RTs) to action probes in the primed action categorization task by kinematic prime (reach-to-drink, reach-to-pour) and action probe (drinking, pouring).

(E) Discrimination performance quantified as the predicted probability of correct choice in the intention discrimination task. In (D) and (E), the histograms show the estimated marginal mean ± SE at the population-level estimated from mixed models fit to single-trial data.

See also Tables S1–S3 and Videos S1 and S2.

We tested this prediction in a primed action categorization task. On each trial, participants observed either a reach-to-drink or reach-to-pour act (kinematic prime) followed by a static image of a person drinking or pouring (action probe). By manipulating the prime-probe relationship, we first established kinematic priming as indexed by faster response times (RTs) to action probes on congruent trials compared with incongruent trials. Using data collected from the same participants in a forced-choice intention discrimination task, we then established the dependency of kinematic priming on intention information encoded and readout in kinematic primes at the single-trial level. Finally, we obtained an independent measure of kinematic priming by demonstrating that prospective gaze control is also primed by single-trial intention information.

RESULTS

Participants (n = 20) first completed a primed action categorization task. To account for inherent variability in movement kinematics, we selected 60 reach-to-grasp acts from a large dataset obtained by tracking and simultaneously filming naive agents performing daily, sequential manipulative actions. In each trial, participants viewed a video featuring a reach-to-drink or reach-to-pour act (kinematic prime), followed by a static image of a person drinking or pouring (action probe) (Figure 1A). To isolate the prospective information encoded in the reach-to-grasp phase of the action, prime stimuli were temporally occluded at the end of the reaching phase, preventing participants from observing the second part of the action (see STAR

CellPress
OPEN ACCESS

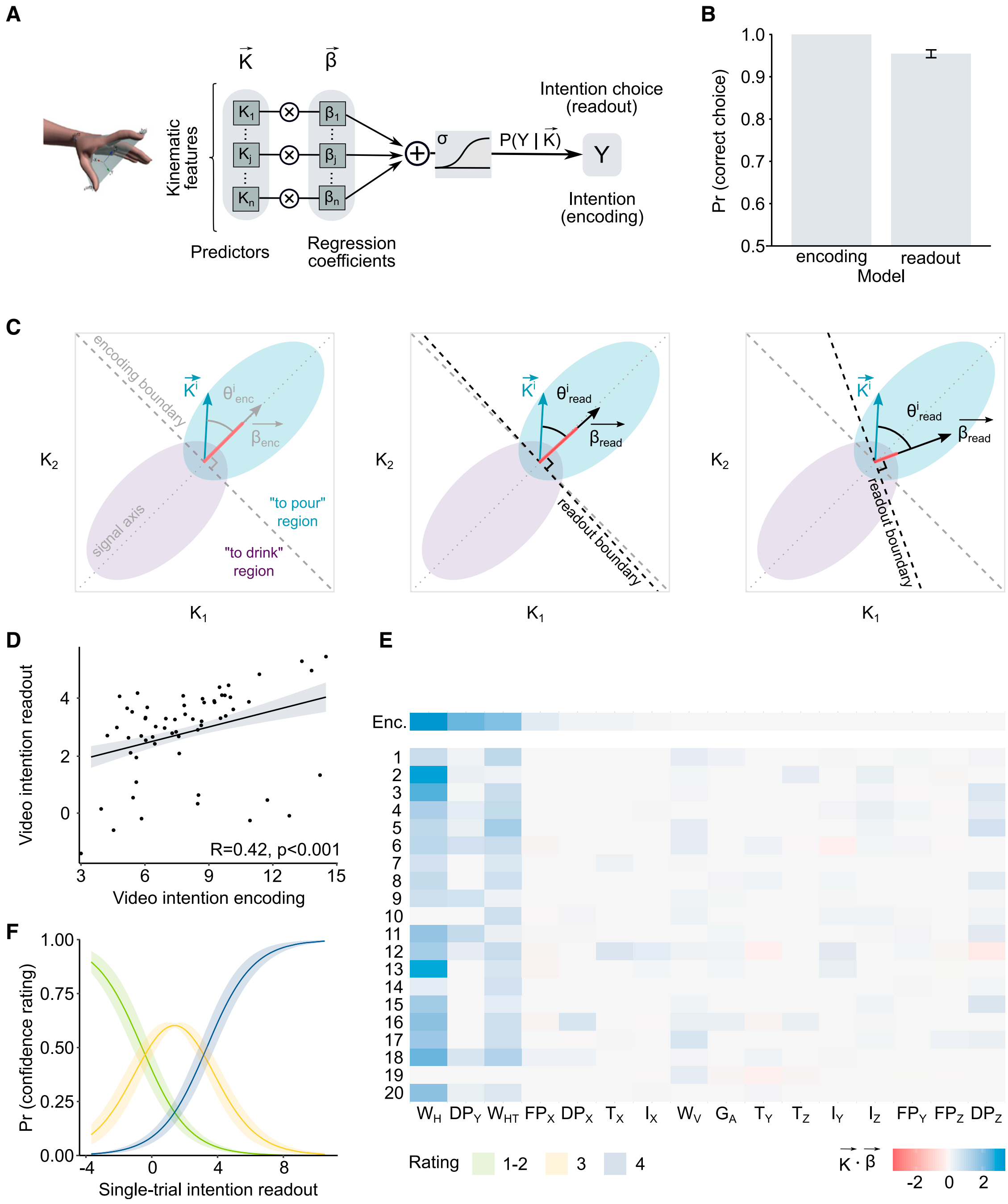

Figure 2. Kinematic coding framework

(A) Schematic of the encoding (readout) model.

(B) Model performance quantified as the predicted probability of correct choice by the encoding (readout) models. The histograms show the estimated marginal mean ± SE at the population-level estimated from mixed models fit to single-trial data.

(C) Sketch of the kinematic encoding (left) and readout (middle and right) models in a simplified two-dimensional kinematic space. Elliptical regions represent the intention-conditional probability distributions in kinematic space. The encoding vector $\overrightarrow{\beta_{enc}}$ is the axis optimally discriminating reach-to-pour from reach-to-drink acts. The alignment of the readout vector $\overrightarrow{\beta_{read}}$ (the axis used by perceivers to discriminate reach-to-pour from reach-to-grasp) relative to the encoding vector determines how efficiently the encoded information is read out.

(D) Spearman correlation between intention readout and intention encoding at the single-prime level. For readout, data points represent the average intention readout across perceivers. The line and shaded region correspond to estimated marginal mean ± SE estimated from a linear model fit to individual prime data.

(E) Color map plotting the contribution of individual kinematic variables to encoding (top) and readout of individual perceivers (bottom, 1–20).

(legend continued on next page)

Methods) (Figure 1B). The task was to categorize the action probe, regardless of the kinematic prime. Because the action probe image was unambiguous, the processing of kinematic prime was not necessary to successfully complete the task. We manipulated the relationship between the kinematic prime and the action probe so that it was either congruent (same intention) or incongruent (different intention) (Videos S1 and S2).

Most demonstrations of priming occur when the probe shares overlapping perceptual or semantic features with the preceding prime. In our task, drinking and pouring action probes share the same degree of perceptual and semantic overlap with reach-to-drink and reach-to-pour primes. Specifically, both reach-to-drink and reach-to-pour primes display an act of reaching for a bottle, with the only difference being the prospective information (to pour, to drink) encoded in the kinematics of the displayed reach. Thus, by design, the manipulation of the congruency between the kinematic prime and the action probe is only relevant to a perceiver who has access to this information. For an observer blind to prospective information, there would be no difference between reach-to-pour and reach-to-drink kinematics, resulting in no facilitation for congruent compared with incongruent trials. Any effect of congruency must therefore be attributed to the use of prospective information encoded in the kinematic prime.

Kinematic priming of response latencies

Mixed-effects statistics to test the effect of kinematic primes (reach-to-drink, reach-to-pour) on RTs to action probes (drinking, pouring) revealed a significant interaction between kinematic prime and action probe, reflecting faster responses to "drinking" (pouring) action probes preceded by reach-to-drink (reach-to-pour) kinematic primes compared with reach-to-pour (reach-to-drink) kinematic primes. p values of statistical comparisons are reported graphically in Figure 1D and numerically in Tables S1–S3. These results show reliable kinematic priming of RTs to both drinking and pouring action probes, indicating that exposure to prospective information in kinematic primes activated a representation of the agent's intention.

Kinematic coding framework

Having established kinematic priming as a behavioral phenomenon, we next investigated its dependency on intention information in kinematic primes. Hand kinematics are high-dimensional, but only a small set of kinematic features encode intention-related information.[6,11] One hypothesis, supported by ideal observer models,[18] is that human perceivers optimally identify and combine these subset of features. Under this encoding-based-priming hypothesis, human computations would approximate the computations of the ideal observer (with some noise) and kinematic priming would be proportional to the information encoded in the kinematics of the observed prime.

Alternatively, perceivers may access intention information in kinematic primes using computations that differ qualitatively from the ideal ones. Our previous work demonstrates that in forced-choice intention discrimination tasks, intention readout computations deviate from optimality.[11] If readout is suboptimal, not all the encoded information is read out. For example, perceivers might read some, but not all the informative features. Under this readout-based priming hypothesis, kinematic priming would be proportional to the information that individual perceivers read in the observed prime, regardless of the overall information encoded and potentially available to an ideal observer.

Testing these hypotheses requires direct measures of how prospective information is encoded and read out at the single-trial level. To obtain these measures, we used our kinematic coding framework.[10–12] This framework was inspired by recent mathematical advancements linking information encoding and readout in neural population activity[19–21] and subsequently adapted to investigate information encoding and readout in movement kinematics.[11,12] Here, we used it to determine encoding and readout computations and measure intention information encoded and read out by individual perceivers in single-prime kinematics.

Kinematic encoding of intention information

We represented the kinematics of each kinematic prime as a vector in the 64-dimensional space of kinematic features (spanning 16 kinematic variables over four time-epochs, see STAR Methods). To determine the subset of kinematic features that encode intention information in individual kinematic primes, we computed the probability that a reaching movement was performed with a given intention (to pour) as a logistic regression of the single-trial kinematic vector of that movement (Figure 2A). Figure 2C shows a geometric sketch of the encoding model in a hypothetical, simplified kinematic space spanning two kinematic features. The encoding boundary defines the boundary that best separates reach-to-pour and reach-to-drink movements. The encoding vector (with components equal to the weights of the encoding logistic regression model) indicates the information axis orthogonal to the encoding boundary, along which changes in kinematics maximally discriminate between reach-to-pour and reach-to-drink. Because the encoding model is trained to classify intention at the optimal level possible, its performance (probability of correct choice) serves as a measure of the available intention information. Additionally, the encoding vector can be used to determine the optimal computations for intention discrimination.

We estimated the intention information encoded in each prime (hereafter, single-trial intention encoding) as the log of the odds of correct intention encoding of that prime. This index is linearly related to the scalar product between the single-trial kinematic vector of that prime and the encoding vector, with sign adjusted so that positive (negative) single-trial intention encoding denoted correct (incorrect) encoding. Larger positive values of single-trial intention encoding indicate larger distance from the encoding boundary and thus greater availability of information for correct discrimination.

(F) Probability of confidence rating in the intention discrimination task as a function of single-trial intention readout. As single-trial intention readout increases, the probability of a lower confidence rating decreases and the probability of a higher confidence rating increases. Lines and shaded regions correspond to estimated marginal means ± SE estimated from the cumulative link mixed model.
See also Figure S2 and Tables S1–S3.

Across trials, intention encoding reached perfect accuracy (Figures 2B and S2D). This indicates that variations in movement kinematics fully specified intention information in each prime. Figures 2E and S2B visualize the contribution of individual kinematic variables to the single-trial intention encoding measured by the scalar product between the encoding vector and the single-trial kinematic vector within the subspace of each variable. In line with Cavallo et al.[6] and Patri et al.,[11] intention information was encoded in a lower-dimensional subspace space spanning three kinematic variables: the height of the wrist (W_H), the horizontal trajectory of the wrist (W_{HT}), and the adduction of hand dorsum (DP_Y) (Figure 2E, top).

Kinematic readout of intention information

The encoding model quantifies the overall intention information encoded in each kinematic prime and available to an ideal observer. To obtain a measure of how human perceivers read such information, 1 h after performing the primed action categorization task, we asked participants to judge the intention of the reaching acts used as kinematic primes in a forced-choice intention discrimination task. Each trial required the perceiver to decide whether the observed reach-to-grasp was performed with the intent to pour or drink. As shown in Figure 1E, perceivers were able to read most, but not all, of the information encoded in kinematic primes. To measure the intention information read in individual primes, we fitted a readout model to the perceivers' intention choices. The readout model computed, separately for each perceiver, the probability of single-trial intention choice, that is, the probability that the perceiver judged the reaching to be performed with the intention to pour, as a logistic regression of the single-trial kinematic vector of the kinematic prime displayed in that trial. Figure 2C sketches the readout model in a hypothetical two-dimensional kinematic space. The readout vector (with components equal to the readout weights of the logistic regression) expresses how the individual perceiver combines features in the kinematic space to discriminate intention. The greater the alignment between the readout vector and the encoding vector, the more efficient the perceiver's readout (for the ideal observer, the readout vector and the encoding vector would coincide). For a given perceiver, we estimated the intention information read in given prime (hereafter, single-trial intention readout) as the log of the odds of correct intention choice. This index is linearly related to the scalar product between the single-trial kinematic vector of that prime and the readout vector that perceiver, with sign adjusted so that positive (negative) single-trial intention readout denoted correct (incorrect) readout of the encoded prospective information. Larger positive values of this index indicate higher correct readout of intention.

Across trials, readout model performance, measured as the probability of correct choice predicted by the readout models, achieved 95% (Figure 2B; for performance of individual readout models and cross-validated performance; see Figures S2A and S2D). This indicates that the readout model accurately captured the dependency of perceivers' intention choices on single-trial movement kinematics. We verified that discrimination accuracies predicted by the model correlated tightly with observed accuracies at the single-subject level (Figure S2F). We further validated the model verifying that human perceivers endorsed with greater confidence choices based on higher intention readout (Figure 2F) but not higher intention encoding (Figure S2E; Tables S1–S3). Overall, these results confirm that our readout model was able to predict how well and how confidently individual perceivers discriminated intention from single-trial kinematics in the intention discrimination task.

Figures 2E and S2C visualize the contribution of individual kinematic variables to single-trial intention readout (measured by the scalar product between the readout vector and the single-trial kinematic vector within the subspace of each variable). Relative to encoding (top), for most perceivers (bottom, 1–20), the subspace of intention readout was reduced to two kinematic variables: W_H and W_{HT} (Figure 2E). Variations in W_H are the most informative regarding intention and, as indicated by a control study, are easily accessible to human perceivers (Figure S1). As shown in Figure 2E, most perceivers read W_H, although to varying degrees. The readout of W_{HT} was sparser and much attenuated, but still present in most participants, whereas only few perceivers read some of the information encoded in DP_Y. To further explore the relationship between encoding and readout at the single-prime level, we plotted the single-trial intention readout against the single-trial intention encoding for each prime. As shown in Figure 2D, a high degree of encoding may lead to varying degrees of readout. This discrepancy is consistent with varying contribution across primes of high- versus low-readout features to encoding. When intention information is encoded in features that are read out effectively similar to W_H, kinematic primes exhibit both high encoding and high readout. When intention information is encoded in features that are not effectively read out, similar to DP_Y, kinematic primes exhibit high encoding but low readout.

Single-trial intention readout predicts kinematic priming of response latencies

The above analyses reveal a disparity between single-trial encoding and readout. We leveraged this disparity to contrast the predictions of the encoding-based-priming hypothesis and the readout-based priming hypothesis. The encoding-based-priming hypothesis predicts a dependency of kinematic priming on single-trial intention encoding, resulting in faster (slower) RTs for congruent (incongruent) trials with high encoding information. The readout-based-priming hypothesis predicts a dependency of kinematic priming on single-trial intention readout, resulting in faster (slower) RTs for congruent (incongruent) trials with high readout information.

Our results (Figures 3A and 3B) support the readout-based priming hypothesis. We found no interaction between single-trial intention encoding and congruency (Figure 3A). However, single-trial intention readout interacted significantly with congruency. Higher intention readout yielded faster RTs on congruent trials and, conversely, slower RTs on incongruent trials (Figure 3B; Table S3). These results indicate a direct, graded influence of single-trial intention readout on kinematic priming, the strength of priming on each trial being proportional to the intention information read on that trial.

To confirm that the predictive power came from differences in single-trial intention readout, we complemented these analyses by performing a median split of kinematic primes based on single-trial intention encoding (readout) and comparing the amount of priming (RTs on incongruent trials minus RTs on congruent

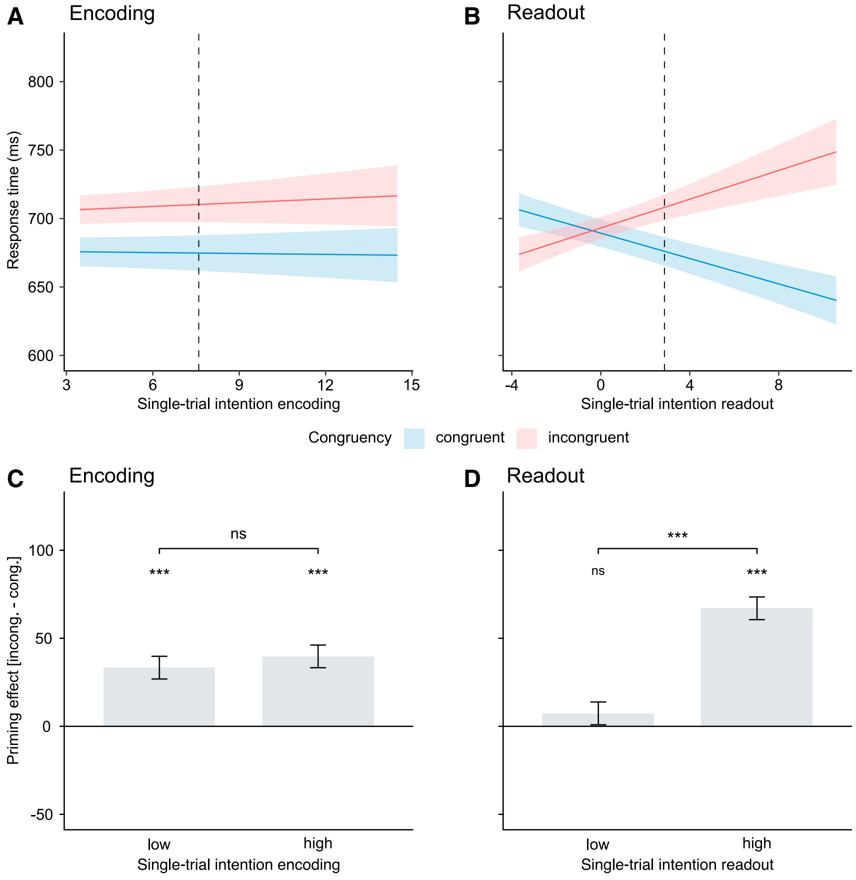

Figure 3. Single-trial intention readout predicts RTs to action probes

(A) RTs to action probes by single-trial intention encoding and congruency.

(B) RTs by single-trial intention readout and congruency.

(C) Priming effect (incongruent-congruent) by low and high encoding information.

(D) Priming effect (incongruent-congruent) by low and high readout information. Mean lines, shaded areas and histograms and error bars represent estimated marginal means ± SE at the population level. Dotted vertical lines (A) and (B) indicate the median value of single-trial intention encoding/readout used for median-split in (C) and (D).

See also Figures S3–S5 and Tables S1–S3.

trials) between high- and low-encoding (readout) trials. Consistent with the readout-based priming hypothesis, kinematic priming was larger and significant for high-readout kinematic primes and smaller and nonsignificant for low-readout kinematic primes (Figure 3D). In contrast, no difference was observed between low- and high-encoding primes (Figure 3C; Table S3).

Another prediction of the readout-based-priming hypothesis is that kinematic primes from which no intention information is read out should not influence responses to action probes, even if they encode intention information. The fitted lines intersecting around zero readout in Figure 3B qualitatively support this prediction. To quantitatively corroborate this observation, we compared kinematic priming of RTs between high-readout trials and zero-readout trials, in which no information was read out from the presented prime in the intention discrimination task. This analysis revealed no kinematic priming for zero-readout trials. As shown in Figure S4, compared with zero-readout trials, RTs were faster on congruent trials and slower on incongruent trials in high-readout trials. This indicates that intention information read in kinematic primes facilitated the processing of congruent probes and hindered the processing of incongruent probes.

Readout computations revealed considerable variability across perceivers (Figure 2E). To establish the relevance of this variability to kinematic priming, we tested whether individual differences in single-trial intention readout could predict individual perceivers' strength of kinematic priming. Intention readout positively correlated with the strength of the kinematic priming effect at the individual level (Figure S3). We also fit readout models to surrogate data obtained pooling trials of all perceivers in order to remove the individuality of readout computations and verified that pooled models could not predict kinematic priming effects (Table S3). These results reinforce the functional relevance of our individualized approach to intention readout, in that they demonstrate that individuality of readout computations matters for kinematic priming.

The dependency of kinematic priming on single-trial intention readout persisted even when controlling for pre-trial fluctuations of alertness as measured by changes in pupil dilation during the pre-trial period[22] (Tables S1–S3). This suggests that trial-to-trial fluctuations in cortical state are unlikely to contribute to kinematic priming.

Collectively, these observations suggest that kinematic priming truly reflects the amount of intention information read by individual perceivers in individual primes, rather than the amount of intention information encoded and potentially available to an ideal observer.

Kinematic priming of initial fixations

To obtain an independent measure of kinematic priming, we examined gaze behavior over the probe image as a function of the previously observed kinematic prime. Gaze control during action perception can be conceptualized as an active process of hypothesis testing where saccadic sampling of regions containing task-relevant information is performed to evaluate competing hypotheses.[23] As such, where a viewer looks reflects a prediction about the most probable location of task-relevant information (see also Henderson[24]). We reasoned that if intention information extracted from kinematic primes is used to predict task-relevant information in action probes, the distribution of initial fixations on the probe image should reflect these predictions. Specifically, the probability of the first fixation being directed to the region containing task-relevant information for the displayed probe (e.g., lower left quadrant when the displayed

CellPress
OPEN ACCESS

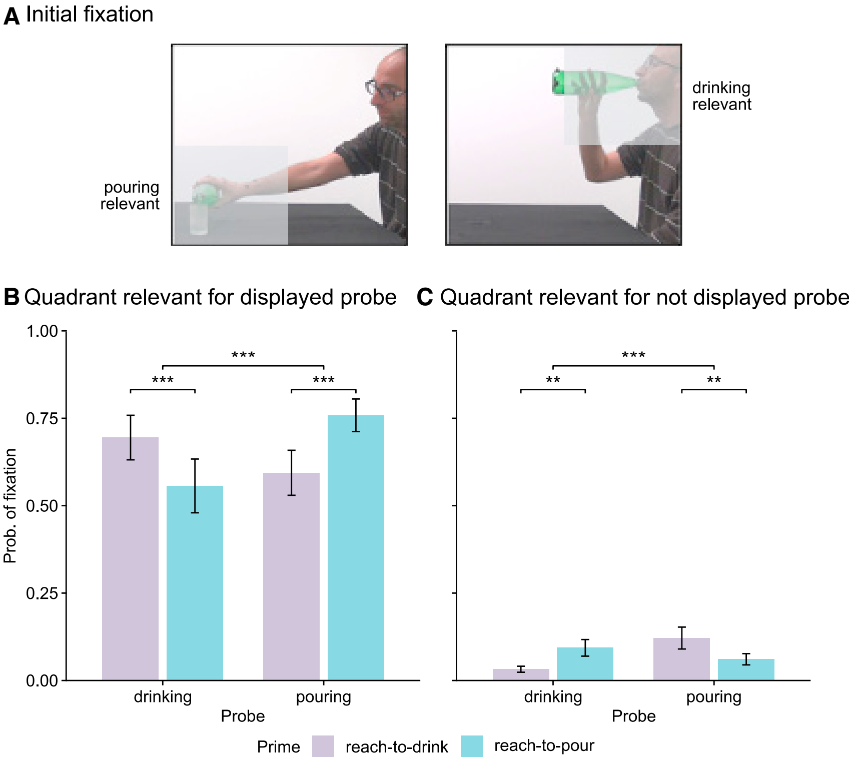

Figure 4. Kinematic priming of initial fixations

(A) Quadrants of task-relevant information in action probes. The shaded area indicates the quadrant of each probe containing task-relevant information.

(B) Probability of initial fixation to the quadrant relevant for the displayed probe by kinematic prime (reach-to-drink, reach-to-pour) and action probe (drinking, pouring).

(C) Probability of initial fixation to the quadrant relevant for the non-displayed probe by kinematic prime (reach-to-drink, reach-to-pour) and action probe (drinking, pouring). Histograms plot the estimated marginal mean ± SE at the population-level estimated from mixed models fit to single-trial data. See also Figure S4 and Tables S1–S3.

probe is pouring) should be higher for congruent trials than for incongruent trials. Conversely, the probability of the first fixation being directed to the region containing task-relevant information for non-displayed probe (upper right quadrant when the displayed probe is pouring) should be higher for incongruent trials than for congruent trials (Figure 4A).

Analyses of initial fixations confirmed these predictions. Initial fixations showed a prime-probe congruency effect driven by a higher probability of initial fixations to the quadrants containing task-relevant information for the displayed probe on congruent compared with incongruent trials (Figure 4B; Table S3). The opposite pattern was observed for initial fixations on the quadrants containing task-relevant for the non-displayed probe: the probability of initial fixation in these quadrants was higher for incongruent than for congruent trials (Figure 4C; Table S3). Taken together, these results indicate that prospective information extracted from kinematic primes guided initial fixations.

As reported in Figure S4A, initial fixations landed on the region predicted to contain task-relevant information approximately 200 ms after the probe was displayed. Incidentally, this observation refutes the idea that participants perform the primed action categorization task by first explicitly identifying the intention of the kinematic prime and then using this information to categorize the intention of the probe image, given that the time required to discriminate kinematic primes in the intention discrimination task exceeds 600 ms (Figure S4A).

Single-trial intention readout predicts kinematic priming of initial fixations

We then investigated whether single-trial intention encoding and readout could predict the probability of initial fixation. Because of the limited number of data points for initial fixations in the quadrants relevant to non-displayed probes, we focused this analysis on quadrants relevant to displayed probes. Single-trial intention encoding did not interact with congruency (Figure 5A; Table S3). However, we found a significant interaction between single-trial intention readout and congruency. Higher intention readout increased the probability of initial fixation on congruent trials and, conversely, decreased (albeit non-significantly), the probability of initial fixation on incongruent trials (Figure 5B; Table S3). Using a median split of single-trial intention encoding (readout) confirmed a dependency of kinematic priming of initial fixation on single-trial intention readout. Kinematic priming of initial fixation was larger for high-readout kinematic primes compared with low-readout kinematic primes (Figure 5D; Table S3), whereas it did not differ between high- and low-encoding primes (Figure 5C). Collectively, these results support the readout-based priming hypothesis and suggest control of gaze truly reflected the information readout at the single-subject, single-trial level.

DISCUSSION

To navigate the social environment, it is often necessary to anticipate the objectives and intentions of other individuals.[1,4] Our research establishes the significance of movement kinematics in this process by presenting evidence for a novel type of priming, which we term kinematic priming. Distinct from visuomotor priming[25,26] or body-part priming,[27] kinematic priming is not induced by action observation—what another person is doing—but rather by action anticipation—what the other person will do next. Using a novel experimental and analytic framework combining kinematic priming with single-trial kinematic coding,[10–12,19] we demonstrate that this anticipation is enabled by the implicit readout of prospective information encoded in movement kinematics and is proportional to the prospective information that individual perceivers read in individual primes.

Readout of intention information varies from perceiver to perceiver and, within the same perceiver, from trial to trial.[11,12] Modeling intention readout with single-subject, single-trial resolution enabled us to predict kinematic priming over a wide range

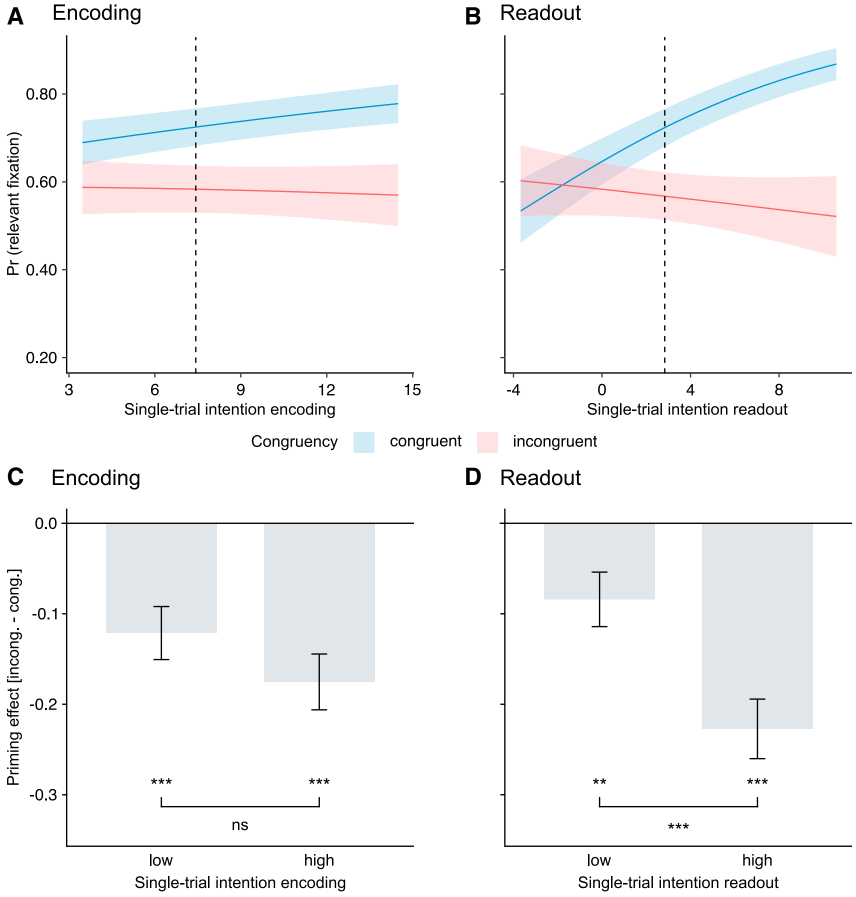

Figure 5. Single-trial intention readout predicts initial fixations

(A) Probability of initial fixation to the region containing the most task-relevant information by single-trial intention encoding.

(B) Probability of initial fixation to the region containing the most task-relevant information by single-trial intention readout.

(C) Priming effect (incongruent-congruent) by low and high encoding information.

(D) Priming effect (incongruent-congruent) by low and high readout information. Conventions are as in Figure 3.

See also Tables S1–S3.

of readout strengths. Higher intention readout yielded faster RTs on congruent trials and slower RTs on incongruent trials. Priming of initial fixations was also modulated by single-trial intention readout, with higher intention readout increasing the probability of fixating selectively on task-relevant regions of the probe in congruent trials, whereas reducing this probability in incongruent trials. These complementary results indicate that prospective information read in kinematic primes influenced both the prediction of the most probable location of task-relevant information (as indexed by initial fixations[23]) and the processing of this information (as indexed by RTs). Together with the observation that prospective information encoded in movement kinematics and potentially available to an ideal observer did not influence responses to action probes, these results provide strong evidence for a direct, graded influence of single-trial kinematic readout on kinematic priming.

We have previously demonstrated that the readout computations by individual perceivers can be revealed by modeling the dependency of explicit intention choices on single-trial movement kinematics in a forced-choice intention discrimination task.[11,12] The readout model identifies the computations by which individual perceivers combine kinematic features to discriminate intention. Our current findings extend this evidence by showing that individual readout computations estimated from a two-alternative forced-choice intention discrimination task can be effectively used to predict the implicit use of prospective information by the same perceiver in a separate task and session (Figures 3B and 5B). These findings highlight the utility of modeling kinematic encoding and readout at the single-subject, single-trial level, and suggest that the estimated computations capture a structural property of kinematic readout geometry of the individual perceiver, which remains temporally stable across sessions and can predict the use of prospective information across tasks. In our design, the two tasks were separated by a relatively short duration (1 h). It remains to be seen to what extent the readout computation remains stable in over days, months, or even years, and whether (and how) experience and tutoring can shape it. Additionally, future research should explore the generalizability of readout computations to multiple outcomes. In our study, although the levels of encoding and readout were continuous in kinematic primes, prediction was between only two possible outcomes: drinking or pouring. For simple perceptual decisions, computations have been demonstrated to be similar for two- and four-choice tasks.[28] This suggests that readout computations supporting action prediction may generalize to three or four outcomes, such as predicting if the person will drink, pour, or pass the bottle to someone else. As the number of possible outcomes increases, perceivers may use contextual information to narrow down the potential outcomes, and then use kinematics to select among them.[1]

At a neural level, there are at least two processing pathways through which kinematics could contact a representation of the action intention. Kinematic priming could be implemented via the ventral pathway linking the middle temporal gyrus to anterior regions of the inferior frontal gyrus.[29] This pathway has been proposed to code a semantic, abstract representation of the action intention. At this level of description, there is no one-to-one mapping between the intention and the action to be performed. Under this hypothesis, kinematic priming would provide access to the most probable intention of the observed reach-to-grasp act (e.g., to drink) at a level of abstraction compatible with several potential available actions (e.g., bringing the bottle to the mouth or drinking from a glass). Alternatively, kinematic

CellPress
OPEN ACCESS

priming may be implemented via the dorsal pathway that connects posterior regions of the inferior frontal gyrus to the anterior portion of the inferior parietal lobule.[8,9,11,29] At this level, the intention maps to a specific action plan of the most probable action to be performed. Under this hypothesis, prospective information read in movement kinematics would prime a concrete representation specifying the spatial metrics of the action to be performed (e.g., lifting the bottle and bringing it to the mouth). We have previously shown that decoding of intention during action observation is most robust from regions of the dorsal pathway[9] and that transient disruption of activity in the anterior portion of the inferior parietal lobule (but not the anterior portion of the inferior frontal gyrus) selectively deteriorated readout computations in a two-alternative forced-intention discrimination task.[11] Here, our analysis of gaze control reveals that perceivers were primed to preselect a specific spatial location on the action probe (e.g., after observing a reach-to-pour prime, they looked at the lower left quadrant, anticipating the interaction of the bottle with the glass in that area). This implies the formation of a concrete representation of the action to be performed. Although these results do not permit any inference about the specific neural mechanisms responsible for kinematic priming, they make a specific neural prediction regarding the involvement of the dorsal pathway. To directly test this hypothesis, future studies could selectively target dorsal and ventral pathway regions with transcranial magnetic stimulation (TMS) to disrupt their function during kinematic priming. Based on our current and previous findings,[11] we would expect that the anterior portion of the inferior parietal lobule is necessary for kinematic priming of intention.

In hierarchical models of action observation, the kinematic level and the intention level are often regarded as separate and independent levels.[30] This has led to the proposal that intention-related variations in movement kinematics are problematic for designing well-controlled measures of intention identification. This is because if different intentions are confounded with different kinematics, it becomes unclear whether choices reflect intention identification or, instead, representation of kinematics without inference of intention.[31] Our findings challenge this view by demonstrating that implicit readout of prospective information in movement kinematics provides perceivers with access to intention representations. In this context, intention-related variations in movement kinematics are not an undesirable confounding factor; rather, they form the basis for inferring intentions. This has implications for action observation models, as well as for models of mindreading, which often assume that mindreading does not entail decoding of observable stimuli.[32]

When perceiving and acting, human perceivers engage in predictive processing at multiple timescales, from milliseconds to seconds and even minutes.[54] Kinematic priming operates on a scale (hundreds of millisecond) that is fundamental for many ecologically important behaviors including, fine motor coordination,[33] embodied decision making,[34,35] social signaling, and transmission.[36–38] The approach described in the current work could be extended to link visual and motor representations[39] and predict individual differences in these domains. Moreover, it could be useful for understanding altered readout computations in clinical disorders such as autism spectrum disorders[12,40] and their relation to difficulties in mindreading in processing real-life social information.[41]

STAR★METHODS

Detailed methods are provided in the online version of this paper and include the following:

- KEY RESOURCES TABLE
- RESOURCE AVAILABILITY
  - Lead contact
  - Materials availability
  - Data and code availability
- EXPERIMENTAL MODEL AND SUBJECT DETAILS
- METHOD DETAILS
  - Primed Action Categorization Task
  - Intention Discrimination Task
  - Kinematic Discrimination Task
- QUANTIFICATION AND STATISTICAL ANALYSIS
  - Kinematic Intersection Framework
  - Assessment of statistical differences

SUPPLEMENTAL INFORMATION

Supplemental information can be found online at https://doi.org/10.1016/j.cub.2023.05.055.

ACKNOWLEDGMENTS

Funded by the European Union under Horizon Europe, grant number 101092889, project SHARESPACE.

AUTHOR CONTRIBUTIONS

C.B., E.S., A.C., and G.B. designed the experiments. S.P. and K.P. designed the analyses with contributions from C.B. and E.S. E.S. and G.B. collected the data. K.P. analyzed the data with contributions from E.S. C.B. and S.P. wrote the first draft of the manuscript with contributions from E.S. and K.P. C.B. and S.P. supervised research and secured funding. All authors revised and approved the final manuscript.

DECLARATION OF INTERESTS

The authors declare no competing interests.

Received: November 24, 2022
Revised: April 6, 2023
Accepted: May 24, 2023
Published: June 19, 2023

REFERENCES

1. Becchio, C., Koul, A., Ansuini, C., Bertone, C., and Cavallo, A. (2018). Seeing mental states: an experimental strategy for measuring the observability of other minds. Phys. Life Rev. *24*, 67–80. https://doi.org/10.1016/j.plrev.2017.10.002.
2. Wispinski, N.J., Gallivan, J.P., and Chapman, C.S. (2020). Models, movements, and minds: bridging the gap between decision making and action. Ann. N. Y. Acad. Sci. *1464*, 30–51. https://doi.org/10.1111/nyas.13973.
3. Gallivan, J.P., Chapman, C.S., Wolpert, D.M., and Flanagan, J.R. (2018). Decision-making in sensorimotor control. Nat. Rev. Neurosci. *19*, 519–534. https://doi.org/10.1038/s41583-018-0045-9.
4. Schultz, J., and Frith, C.D. (2022). Animacy and the prediction of behaviour. Neurosci. Biobehav. Rev. *140*, 104766. https://doi.org/10.1016/j.neubiorev.2022.104766.
5. Forstmann, B.U., Ratcliff, R., and Wagenmakers, E.J. (2016). Sequential sampling models in cognitive neuroscience: advantages, applications,

and extensions. Annu. Rev. Psychol. *67*, 641–666. https://doi.org/10.1146/annurev-psych-122414-033645.

6. Cavallo, A., Koul, A., Ansuini, C., Capozzi, F., and Becchio, C. (2016). Decoding intentions from movement kinematics. Sci. Rep. *6*, 37036. https://doi.org/10.1038/srep37036.
7. Koul, A., Soriano, M., Tversky, B., Becchio, C., and Cavallo, A. (2019). The kinematics that you do not expect: integrating prior information and kinematics to understand intentions. Cognition *182*, 213–219. https://doi.org/10.1016/j.cognition.2018.10.006.
8. Soriano, M., Cavallo, A., D'Ausilio, A., Becchio, C., and Fadiga, L. (2018). Movement kinematics drive chain selection toward intention detection. Proc. Natl. Acad. Sci. USA *115*, 10452–10457. https://doi.org/10.1073/pnas.1809825115.
9. Koul, A., Cavallo, A., Cauda, F., Costa, T., Diano, M., Pontil, M., and Becchio, C. (2018). Action observation areas represent intentions from subtle kinematic features. Cereb. Cortex *28*, 2647–2654. https://doi.org/10.1093/cercor/bhy098.
10. Becchio, C., Pullar, K., and Panzeri, S. (2021). Costs and benefits of communicating vigor. Behav. Brain Sci. *44*, e124. https://doi.org/10.1017/S0140525X21000200.
11. Patri, J.-F., Cavallo, A., Pullar, K., Soriano, M., Valente, M., Koul, A., Avenanti, A., Panzeri, S., and Becchio, C. (2020). Transient disruption of the inferior parietal lobule impairs the ability to attribute intention to action. Curr. Biol. *30*, 4594–4605.e7. https://doi.org/10.1016/j.cub.2020.08.104.
12. Montobbio, N., Cavallo, A., Albergo, D., Ansuini, C., Battaglia, F., Podda, J., Nobili, L., Panzeri, S., and Becchio, C. (2022). Intersecting kinematic encoding and readout of intention in autism. Proc. Natl. Acad. Sci. USA *119*, e2114648119, https://doi.org/10.1073/pnas.2114648119.
13. Catmur, C. (2015). Understanding intentions from actions: direct perception, inference, and the roles of mirror and mentalizing systems. Conscious. Cogn. *36*, 426–433. https://doi.org/10.1016/j.concog.2015.03.012.
14. Schacter, D.L., Dobbins, I.G., and Schnyer, D.M. (2004). Specificity of priming: a cognitive neuroscience perspective. Nat. Rev. Neurosci. *5*, 853–862. https://doi.org/10.1038/nrn1534.
15. Cooper, L.A., Schacter, D.L., Ballesteros, S., and Moore, C. (1992). Priming and recognition of transformed three-dimensional objects: effects of size and reflection. J. Exp. Psychol. Learn. Mem. Cogn. *18*, 43–57. https://doi.org/10.1037//0278-7393.18.1.43.
16. Biederman, I., and Cooper, E.E. (1991). Priming contour-deleted images: evidence for intermediate representations in visual object recognition. Cogn. Psychol. *23*, 393–419. https://doi.org/10.1016/0010-0285(91)90014-f.
17. Naccache, L., and Dehaene, S. (2001). The priming method: imaging unconscious repetition priming reveals an abstract representation of number in the parietal lobes. Cereb. Cortex *11*, 966–974. https://doi.org/10.1093/cercor/11.10.966.
18. Chin, B.M., and Burge, J. (2020). Predicting the partition of behavioral variability in speed perception with naturalistic stimuli. J. Neurosci. *40*, 864–879. https://doi.org/10.1523/JNEUROSCI.1904-19.2019.
19. Panzeri, S., Harvey, C.D., Piasini, E., Latham, P.E., and Fellin, T. (2017). Cracking the neural code for sensory perception by combining statistics, intervention, and behavior. Neuron *93*, 491–507. https://doi.org/10.1016/j.neuron.2016.12.036.
20. Valente, M., Pica, G., Bondanelli, G., Moroni, M., Runyan, C.A., Morcos, A.S., Harvey, C.D., and Panzeri, S. (2021). Correlations enhance the behavioral readout of neural population activity in association cortex. Nat. Neurosci. *24*, 975–986. https://doi.org/10.1038/s41593-021-00845-1.
21. Panzeri, S., Moroni, M., Safaai, H., and Harvey, C.D. (2022). The structures and functions of correlations in neural population codes. Nat. Rev. Neurosci. *23*, 551–567. https://doi.org/10.1038/s41583-022-00606-4.
22. Reimer, J., McGinley, M.J., Liu, Y., Rodenkirch, C., Wang, Q., McCormick, D.A., and Tolias, A.S. (2016). Pupil fluctuations track rapid changes in adrenergic and cholinergic activity in cortex. Nat. Commun. *7*, 13289. https://doi.org/10.1038/ncomms13289.
23. Donnarumma, F., Costantini, M., Ambrosini, E., Friston, K., and Pezzulo, G. (2017). Action perception as hypothesis testing. Cortex *89*, 45–60. https://doi.org/10.1016/j.cortex.2017.01.016.
24. Henderson, J.M. (2017). Gaze control as prediction. Trends Cogn. Sci. *21*, 15–23. https://doi.org/10.1016/j.tics.2016.11.003.
25. Fagioli, S., Ferlazzo, F., and Hommel, B. (2007). Controlling attention through action: observing actions primes action-related stimulus dimensions. Neuropsychologia *45*, 3351–3355. https://doi.org/10.1016/j.neuropsychologia.2007.06.012.
26. Helbig, H.B., Steinwender, J., Graf, M., and Kiefer, M. (2010). Action observation can prime visual object recognition. Exp. Brain Res. *200*, 251–258. https://doi.org/10.1007/s00221-009-1953-8.
27. Springer, A., Brandstädter, S., and Prinz, W. (2013). Dynamic simulation and static matching for action prediction: evidence from body part priming. Cogn. Sci. *37*, 936–952. https://doi.org/10.1111/cogs.12044.
28. Churchland, A.K., Kiani, R., and Shadlen, M.N. (2008). Decision-making with multiple alternatives. Nat. Neurosci. *11*, 693–702. https://doi.org/10.1038/nn.2123.
29. Kilner, J.M. (2011). More than one pathway to action understanding. Trends Cogn. Sci. *15*, 352–357. https://doi.org/10.1016/j.tics.2011.06.005.
30. Grafton, S.T., and Hamilton, A.F. (2007). Evidence for a distributed hierarchy of action representation in the brain. Hum. Mov. Sci. *26*, 590–616. https://doi.org/10.1016/j.humov.2007.05.009.
31. Thompson, E.L., Bird, G., and Catmur, C. (2019). Conceptualizing and testing action understanding. Neurosci. Biobehav. Rev. *105*, 106–114. https://doi.org/10.1016/j.neubiorev.2019.08.002.
32. Apperly, I.A. (2019). How is mindreading really like reading? Behav. Brain Sci. *42*, e170. https://doi.org/10.1017/S0140525X19001031.
33. Ansuini, C., Podda, J., Battaglia, F.M., Veneselli, E., and Becchio, C. (2018). One hand, two hands, two people: prospective sensorimotor control in children with autism. Dev. Cogn. Neurosci. *29*, 86–96. https://doi.org/10.1016/j.dcn.2017.02.009.
34. Gordon, J., Maselli, A., Lancia, G.L., Thiery, T., Cisek, P., and Pezzulo, G. (2021). The road towards understanding embodied decisions. Neurosci. Biobehav. Rev. *131*, 722–736. https://doi.org/10.1016/j.neubiorev.2021.09.034.
35. Turri, G., Cavallo, A., Romeo, L., Pontil, M., Sanfey, A., Panzeri, S., and Becchio, C. (2022). Decoding social decisions from movement kinematics. iScience *25*, 105550. https://doi.org/10.1016/j.isci.2022.105550.
36. Eijk, L., Rasenberg, M., Arnese, F., Blokpoel, M., Dingemanse, M., Doeller, C.F., Ernestus, M., Holler, J., Milivojevic, B., Özyürek, A., et al. (2022). The CABB dataset: A multimodal corpus of communicative interactions for behavioural and neural analyses. NeuroImage *264*, 119734. https://doi.org/10.1016/j.neuroimage.2022.119734.
37. McEllin, L., Sebanz, N., and Knoblich, G. (2018). Identifying others' informative intentions from movement kinematics. Cognition *180*, 246–258. https://doi.org/10.1016/j.cognition.2018.08.001.
38. Pezzulo, G., Donnarumma, F., Dindo, H., D'Ausilio, A., Konvalinka, I., and Castelfranchi, C. (2019). The body talks: sensorimotor communication and its brain and kinematic signatures. Phys. Life Rev. *28*, 1–21. https://doi.org/10.1016/j.plrev.2018.06.014.
39. Giese, M.A., and Rizzolatti, G. (2015). Neural and computational mechanisms of action processing: interaction between visual and motor representations. Neuron *88*, 167–180. https://doi.org/10.1016/j.neuron.2015.09.040.
40. Edey, R., Cook, J., Brewer, R., Johnson, M.H., Bird, G., and Press, C. (2016). Interaction takes two: typical adults exhibit mind-blindness towards those with autism spectrum disorder. J. Abnorm. Psychol. *125*, 879–885. https://doi.org/10.1037/abn0000199.
41. Redcay, E., and Schilbach, L. (2019). Using second-person neuroscience to elucidate the mechanisms of social interaction. Nat. Rev. Neurosci. *20*, 495–505. https://doi.org/10.1038/s41583-019-0179-4.

CellPress
OPEN ACCESS

42. World Medical Association (2013). World Medical Association Declaration of Helsinki: ethical principles for medical research involving human subjects. JAMA *310*, 2191–2194. https://doi.org/10.1001/jama.2013.281053.

43. Winn, M.B., Wendt, D., Koelewijn, T., and Kuchinsky, S.E. (2018). Best practices and advice for using pupillometry to measure listening effort: an introduction for those who want to get started. Trends Hear. *22*. 2331216518800869. https://doi.org/10.1177/2331216518800869.

44. Zou, H., and Hastie, T. (2005). Regularization and variable selection via the elastic net. J. R. Stat. Soc. B *67*, 301–320. https://doi.org/10.1111/j.1467-9868.2005.00503.x.

45. Friedman, J., Hastie, T., and Tibshirani, R. (2010). Regularization paths for generalized linear models via coordinate descent. J. Stat. Softw. *33*, 1–22.

46. Hastie, T., Tibshirani, R., and Wainwright, M. (2015). Statistical Learning with Sparsity: the Lasso and Generalizations (Chapman and Hall/CRC).

47. Ng, V.K.Y., and Cribbie, R.A. (2017). Using the gamma generalized linear model for modeling continuous, skewed and heteroscedastic outcomes in psychology. Curr. Psychol. *36*, 225–235. https://doi.org/10.1007/s12144-015-9404-0.

48. Lo, S., and Andrews, S. (2015). To transform or not to transform: using generalized linear mixed models to analyse reaction time data. Front. Psychol. *6*, 1171. https://doi.org/10.3389/fpsyg.2015.01171.

49. Bates, D., Mächler, M., Bolker, B.M., and Walker, S.C. (2015). Fitting linear mixed-effects models using lme4. J. Stat. Softw. *67*, 1–48. https://doi.org/10.18637/jss.v067.i01.

50. Schad, D.J., Vasishth, S., Hohenstein, S., and Kliegl, R. (2020). How to capitalize on a priori contrasts in linear (mixed) models: A tutorial. J.MemL *110*, 104038. https://doi.org/10.1016/j.jml.2019.104038.

51. McCabe, C.J., Halvorson, M.A., King, K.M., Cao, X., and Kim, D.S. (2022). Interpreting interaction effects in generalized linear models of nonlinear probabilities and counts. Multivariate Behav. Res. *57*, 243–263. https://doi.org/10.1080/00273171.2020.1868966.

52. Mize, T. (2019). Best practices for estimating, interpreting, and presenting nonlinear interaction effects. Sociol. Sci. *6*, 81–117. https://doi.org/10.15195/v6.a4.

53. Mood, C. (2010). Logistic regression: why we cannot do what we think we can do, and what we can do about It. Eur. Sociol. Rev. *26*, 67–82. https://doi.org/10.1093/esr/jcp006.

54. Kurby, C.A., and Zacks, J.M. (2022). Priming of movie content is modulated by event boundaries. J. Exp. Psychol. Learn. Mem. Cogn. *48*, 1559–1570. https://doi.org/10.1037/xlm0001085.

STAR★METHODS

KEY RESOURCES TABLE

REAGENT or RESOURCE	SOURCE	IDENTIFIER
Deposited Data		
Data supporting main findings	This paper	Mendeley Data: https://data.mendeley.com/datasets/m6s3r6fzzs/ https://doi.org/10.17632/m6s3r6fzzs.2
Software and Algorithms		
Adobe Premiere Pro	Adobe	https://www.adobe.com/products/premiere.html RRID:SCR_021315
Eyelink 1000 Eye Tracking System	SR Research, Ontario, Canada	https://www.sr-research.com/software/ RRID:SCR_009602
R (Version 4.0.5)	R Project for Statistical Computing	https://www.R-project.org/ RRID:SCR_001905
R package: glmnet (Version 4.1-3)	Thompson et al.[31]	https://cran.r-project.org/web/packages/glmnet/index.html RRID:SCR_015505
R package: lme4 (Version 1.1-27.1)	Turri et al.[35]	https://cran.r-project.org/web/packages/lme4/index.html RRID:SCR_015654
R package: ordinal (Version 2019.12-10)	N/A	https://cran.r-project.org/package=ordinal RRID:SCR_022856
R package: emmeans (Version 1.7.2)	N/A	https://cran.r-project.org/web/packages/emmeans/index.html RRID:SCR_018734
Matlab (Version R2019b)	MathWorks Inc.	http://www.mathworks.com/products/matlab/ RRID:SCR_001622

RESOURCE AVAILABILITY

Lead contact

Further information and requests for resources should be directed and will be fulfilled by the lead contact, Cristina Becchio (c.becchio@uke.de).

Materials availability

This study did not generate new unique reagents or materials.

Data and code availability

The data supporting the main findings of this study are available for download at the following link (https://data.mendeley.com/datasets/m6s3r6fzzs/). The code supporting the main findings of this study is based on public available tools listed in the key resources table. Custom functions inputting data to toolboxes will be made available by the lead contact upon reasonable request.

EXPERIMENTAL MODEL AND SUBJECT DETAILS

Twenty participants (9 females, 11 males, mean age 31, range 21-40 years) took part in the experiment. All participants were right-handed, had normal or corrected-to-normal vision and were naïve to the purpose of the experiment. None of them declared any history of psychiatric or neurological diseases. The research was approved by the local ethical committee (ASL 3 Genovese) and was carried out in accordance with the principles of the revised Helsinki Declaration.[42] All participants provided written informed consent and received monetary compensation in return for their participation.

METHOD DETAILS

All participants completed a primed action categorization task and, one hour later, an intention discrimination task on the stimuli used as kinematic primes in the primed action categorization task. To avoid any influence of intention discrimination on kinematic priming, all participants performed the primed action categorization task first.

Primed Action Categorization Task

Experimental design

Kinematic primes consisted of 30 videos of grasp-to-pour movements and 30 videos of grasp-to-drink movements. On each trial, participants observed either a grasp-to-drink or grasp-to-pour prime, followed by a pouring or drinking action probe (Figure 1A). The action probe consisted of a photograph of a male agent drinking or pouring. We manipulated the relationship between the kinematic prime and the action probe on the intention dimension so that it was congruent in 75% of the trials and incongruent in 25% of the trials.

Procedure and apparatus

Each trial began with the presentation of a central fixation cross for 4000 ms, then a kinematic prime was presented, followed by an action probe. Participants were asked to indicate via a button press whether the agent depicted in the action probe drank or poured (right key = "drink"; left key = "pour", counterbalanced across participants). The action probe remained on screen for a maximum of 3000 ms or until participant's response and was followed by a fixation cross screen of 2000 ms. The session began with a practice block of 8 trials (6 congruent and 2 incongruent). Participants then performed three blocks of 80 trials (60 congruent, 20 incongruent trials), for a total of 240 trials. No feedback was provided. Stimuli were presented on a 21.5-inch LCD monitor with a resolution of 1,920 × 1,080 pixels (refresh rate: 60 Hz), viewed from a distance of 100 cm, with the head stabilized by a chin rest. Stimulus presentation, timing, and randomization were controlled using Experiment Builder software (SR Research, Ontario, Canada).

Kinematic primes. Acquisition and analysis

Reach-to-grasp movements used as kinematic primes were chosen from a large dataset obtained by tracking and simultaneously filming 17 participants performing four naturalistic sequential activities: i) reaching for, grasping, lifting a bottle, and pouring water into a glass; ii) reaching for, grasping, lifting a bottle, and taking a sip from it; iii) reaching for, grasping, lifting, and placing the bottle into a box; iv) reaching for, grasping, lifting, and passing the bottle to a co-experimenter. Each participant completed 2 blocks of 40 trials, with 10 consecutive trials for each action sequence in each block. The order of conditions was counterbalanced across participants. In i) and ii), a co-experimenter refilled the bottle on each trial. Detailed apparatus and procedures for motion-tracking are described in Cavallo et al.[6] In brief, each participant was outfitted with 20 lightweight retro-reflective hemispheric markers (4 mm in diameter). Reach-to-grasp movements were tracked using a near-infrared camera motion capture system with nine cameras (Vicon Motion Systems Ltd, Oxford, UK; frame rate: 100 Hz) and concurrently filmed from a lateral viewpoint using a digital video camera (Sony Handycam 3D, 25 frames/sec; Sony Corporation, Tokyo, Japan). Computation of kinematic variables was based on Cavallo et al.[6] and followed identical procedures. We used custom software (Matlab; MathWorks Inc., Natick, MA) to compute two sets of kinematic variables of interest: F_{global} and F_{local} variables. F_{global} variables were expressed with respect to the global frame of reference, i.e., the frame of reference of the motion capture system. Within this frame of reference, we computed the following variables:

- wrist velocity, defined as the module of the three-dimensional velocity vector of the wrist marker (mm/sec);
- wrist height, defined as the z-component of the wrist marker (mm);
- wrist horizontal trajectory, defined as the x-component (transverse component) of the wrist marker (mm);
- grip aperture, defined as the distance between the marker placed on thumb tip and the one placed on the tip of the index finger (mm).

To provide a better characterization of the hand joint movements, the second set of variables was expressed with respect to a local frame of reference centred on the hand (i.e., F_{local}). Within F_{local}, we computed the following variables:

- x-, y-, and z-thumb defined as x-, y- and z-coordinates for the thumb with respect to F_{local} (mm);
- x-, y-, and z-index defined as x-, y- and z-coordinates for the index with respect to F_{local} (mm);
- x-, y-, and z-finger plane defined as x-, y- and z-components of the thumb-index plane, i.e., the three-dimensional components of the vector that is orthogonal to the plane, providing information about the abduction/adduction movement of the thumb and index finger irrespective of the effects of wrist rotation and of finger flexion and extension;
- x-, y-, and z-dorsum plane defined as x-, y- and z-components of the radius-phalanx plane, providing information about the abduction, adduction, and rotation of the hand dorsum irrespective of the effects of wrist rotation.

All variables were calculated only considering the reach-to-grasp phase of the movement, from 'reach onset' (i.e., the first time point at which the wrist velocity crossed a 20 mm/s threshold) to 'reach offset' (i.e., the first time point at which the wrist velocity dropped below a 20 mm/s threshold).

Kinematic prime: selection and post-processing

From the above dataset, we selected 30 reach-to-pour and 30 reach-to-drink movements. Acts were selected based on previous data in our laboratory[6–8] with a target discrimination accuracy of about 75% in the intention discrimination task. Movement duration

(mean ± SEM = 1.04 ± 0.02 s, range = 0.84 to 1.36 s) did not differ between intentions ($t_{(58)}$ = 1.36; p = 0.18). The 60 unique video clips corresponding to the selected movements were edited using Adobe Premiere Pro CS6 (Adobe Systems Software Ltd, Dublin, Ireland; mp4 format, disabled audio, 25 frames/s, resolution 1280 x 800) so that each clip started at reach onset and ended at reach offset. To allow participants enough time to focus on initiation of the movement, 9, 11, or 13 static frames (corresponding to 360 ms, 440 ms and 520 ms) were randomly added at the beginning of each video.

Eye tracking apparatus and paradigm

Eye movements were monitored using an EyeLink 1000 Plus desk-mounted eye tracker (SR Research, Ontario, Canada), which uses infrared pupil detection and corneal reflection to track eye movements. Eye movements and pupil diameter were recorded monocularly from the participants' right eyes at 1000 Hz. Participants' eye movements were calibrated and validated using a nine-point calibration fixation sequence at the beginning of the experimental session. Eye movement data were analysed using the Data Viewer software (version 4.1.211, SR Research, Ontario, Canada). Initial fixations were defined as average gaze position during periods where the change in recorded gaze direction was smaller than 0.1°, eye movement velocity was below 30°/s, and acceleration was below 8000°/s^2. To assess the influence of kinematic priming on gaze control, we divided each action probe into four equal quadrants and identified the quadrant that contained most task-relevant information (top right quadrant with mouth-bottle interaction for drinking, bottom left quadrant with bottle-glass interaction for pouring, Figure 4A).

To examine the possible influence of trial-to-trial fluctuations of alertness on kinematic priming, we computed the averaged baseline pupil diameter in the one second preceding the display of the kinematic prime. Pupil diameter signals were pre-processed with standard techniques.[43] Blinks were treated with linear interpolation, and the resulting pupil traces were smoothed with a first-order 10 Hz low-pass-filter.

Intention Discrimination Task

One hour after completing the primed action categorization task, participants performed a forced-choice intention discrimination task on the stimuli used as kinematic primes in the primed action categorization task (Figure 1A). Task structure conformed to a one-interval two-alternative forced-choice discrimination task. Each trial started with the presentation of a central fixation cross for 4000 ms, then a reaching act was presented, followed by a fixation cross. Participants were asked to indicate via button press (right key = "pour"; left key = "drink", counterbalanced across participants) the intention of the observed reaching act. 2000 ms after response, the screen prompted participants to rate the confidence of their decision on a four-point scale by pressing a key (from 1 = least confident to 4 = most confident). Participants were encouraged to use the entire confidence scale. If no response was given within 3000 ms, the next trial was presented. No feedback was provided to participants at any stage of the experiment. The session began with a practice block of 8 trials (4 trials for each intention). Participants then performed 4 blocks of 60 trials, for a total of 240 trials. Each video was viewed once in each block. Stimulus presentation, timing and randomization procedure were controlled using Experiment Builder software (SR Research, Ontario, Canada).

Kinematic Discrimination Task

We performed a control analysis designed to test human perceptual accessibility to kinematic features observed in the intention discrimination task (Figure S1A). Specifically, we tested 8 new participants to assess their ability to discriminate wrist height. Task structure conformed to a two alternative forced choice (2AFC) design. The kinematic discrimination task included the same stimuli as the intention discrimination task, except that participants were asked to indicate the interval containing the grasp with higher peak vertical height of the wrist. The kinematic discrimination task consisted of two blocks of 30 trials. Each trial displayed two reach-to-grasp acts in two consecutive temporal intervals. Each trial started with the presentation of a green central fixation cross for 1500 ms. Then, the first grasping act was presented followed by an inter-stimulus interval of 500 ms, after which the second grasping act was presented. After the end of the second video, the screen prompted participants to indicate the interval (first or second) containing the higher wrist height by pressing a key. The prompt screen was displayed until response or for a maximum duration of 3000 ms. After response, participants were requested to rate the confidence of their choice on a four-point scale by pressing a key. Pairing of videos was randomized across trials and participants. Participants began the session by performing a practice block of 4 trials before the main experimental task. No feedback was provided. Stimulus presentation, timing and randomization was controlled using E-prime V2.0 software (Psychology Software Tools, Pittsburgh, PA).

QUANTIFICATION AND STATISTICAL ANALYSIS

Kinematic Intersection Framework

Single-trial kinematic vector

The kinematics of the hand are higher dimensional than those of other effectors. To establish which dimensions are relevant for the encoding (and readout) of intention information, we started with a high-dimensional model space and then used regularized regression with penalty terms and cross-validated methods, to define the kinematic subspace that captures intention information. As described in Patri et al.,[11] we averaged the 16 kinematic variables of interest over 4 time epochs of 25% of the normalized movement time (0-25%, 25-50%, 50-75%, and 75-100% of movement duration defined from reach onset to reach offset). For each trial, we created a 64-dimensional vector defined by the 16 kinematic variables over the 4 time epochs (64 kinematic features). We verified

that increasing the number of time epochs to 6 and 8 did not significantly increase the performance of the encoding or readout models ($p > 0.3$ for all comparisons).

Kinematic encoding and readout models

To quantify the dependence of intention on trial-to-trial variations in movement kinematics (i.e., kinematic encoding of intention information), we trained a logistic regression model to predict the probability that a reach was performed with the intention 'to pour' as a function of the single-trial kinematic vector (Figure 2A). The logistic regression assumes that the log of the odds of to pour vs to drink depends linearly on the kinematics. Thus, the kinematic encoding model expressed single-trial probability Y of intention 'to pour' as a sigmoid transformation of a weighted sum of the components of the single-trial kinematic vector $\overrightarrow{K}$, as expressed by the following equation:

$$P\left([Y = 'to - pour'] \middle| \overrightarrow{K}\right) = \sigma\left(\overrightarrow{\beta} \cdot \overrightarrow{K} + \beta_0\right)$$
$$P\left([Y = 'to - drink'] \middle| \overrightarrow{K}\right) = 1 - P\left([Y = 'to - pour'] \middle| \overrightarrow{K}\right) \quad \text{(Equation 1)}$$

where σ is the sigmoid function, $\overrightarrow{\beta}$ is the vector containing the values of the regression coefficients of each kinematic feature, and β_0 is the bias, kinematic independent, term.

Similarly, to quantify the dependence of intention choice on trial-to-trial variations in movement kinematics (i.e., kinematic readout of intention information), we trained, separately for each perceiver, a logistic regression classifier to predict the single-trial probability Y of intention choice 'to pour' as a function of the single-trial kinematic vector.

Training kinematic encoding and readout models

Training and evaluation were performed in a similar manner for kinematic encoding and readout models. To avoid penalizing predictors with larger ranges of values, we z-scored single-trial kinematic vectors within each model. Models were trained using elastic-net regularization, with a value of $\alpha = 0.95$ for the elastic net parameter to provide sparse solutions in parameter space.[44] To confirm that the pattern of readout weights was robust to the choice of the elastic net hyper-parameter α, we computed for each participant the correlation between the readout weights for $\alpha = 0.95$ with the readout weights obtained with values of α ranging from 0.5 to 1. Correlation values decreased with α values but remained higher than 0.9 for all $\alpha > 0.5$. The parameter λ, which controls the strength of the regularization term, was estimated for each model using leave-one-video-out cross-validation. For each model, we retained the value λ_{min} associated to the minimum mean cross-validated error. Models were then trained on all trials with the retained regularization term. Encoding models were trained on the 60 reaching acts. Readout models were trained, separately for each participant, on 240 trials (resulting from four repetitions of each the 60 videos in the intention discrimination tasks). Logistic regression was implemented using R package "glmnet"[45] (https://CRAN.R-project.org/package=glmnet, 4.1–3).

In the main text, we report the results obtained by applying this training procedure as it gives only one set of regression coefficients per analysed case, and it is therefore easier to interpret. However, as shown in Figure S2D, results remained highly significant ($p < 0.001$) using nested leave-one-video-out cross validation on the entire procedure.

Evaluation of model performance

To quantify model performance, we computed the most likely value of Y for each trial by taking the argmax over Y of $P(Y | \overrightarrow{K})$ in Equation 1. This function returns an estimate of the model prediction (prediction of the actual intention for the encoding model; prediction of observer's choice for the readout model) on each trial. We then quantified model performance as the fraction of correct predictions over trials. The chance-level null-hypothesis distribution for readout model performance was created by fitting the model after randomly permuting across trials the observer's choice labels.

Verification of the statistical significance of non-zero regression coefficients

To verify the statistical significance of non-zero regression coefficients,[46] we did a permutation test in which a null hypothesis distribution of regression weights was obtained after random permutations of the trial labels. We took the absolute value of each individual regression coefficient obtained in the permuted dataset to build a distribution of absolute values of regression coefficient expected under the null-hypothesis of no relationship between the kinematics and the variable Y. We verified that all non-zero beta coefficients had an absolute value that exceeded the 95th percentile of this null-hypothesis distribution.

Computation of single-trial encoding and readout of intention information

We computed an index of intention information encoded in a single trial (termed single-trial kinematic encoding) as the log of the odds of correct encoding. This index equals the argument of the sigmoid function in the logistic regression model (Equation 1), with sign adjusted so that positive (negative) single-trial intention encoding denoted correct (incorrect) encoding. Thus, its magnitude directly quantifies the confidence (the log of the odds of the two alternatives) of the intention classification performed by the encoding model in that trial. When the kinematic-independent bias term β_0 in Equation 1 is null, this index equals the sign-adjusted scalar product between the single-trial kinematic vector $\overrightarrow{K}$ of that prime and the encoding vector $\overrightarrow{\beta_{enc}}$ (with components equal to the encoding weights). Similarly, we computed single-trial intention readout as the log of the odds of correct readout. As for encoding, its magnitude relates to the confidence of the intention choice classification by the readout model. When the bias β_0 is null, the single trial intention readout equals the sign-adjusted scalar product between the single-trial kinematic vector $\overrightarrow{K}$ and the readout vector $\overrightarrow{\beta_{read}}$ (with components equal to the readout weights).

Contribution of individual kinematic variables to intention encoding and readout
The colour map in Figure 2E visualizes the contribution of individual kinematic variables to intention encoding and readout. Single variable contribution to encoding (readout) performance was computed as the scalar product between the kinematic vector and the encoding (readout) vector calculated within the feature subspace formed by the features of the considered kinematic variable (e.g., 25%, 50%, 75% and 100% of movement time for W_H). Positive (negative) values of this index imply a positive (negative) contribution of the variable toward enhancing (decreasing) discrimination performance. Bar plots of the contribution of individual kinematic variables to encoding and readout are shown in Figures S2B and S2C, respectively.

Pooled readout models
For this control analysis, we fit readout models on surrogate data obtained pooling trials of all perceivers. To ensure fully comparable model training and testing conditions in terms of data numerosity, we resampled the pooled-trials dataset to create 20 surrogate participants that had their data randomly sampled (eliminating the identity of the perceivers the trials came from) with exactly the same number of trials per participant as in the original data. The pooled readout models were then fit as described in the *Training kinematic encoding and readout models* section. The results of this analysis are presented in Tables S1–S3.

Null readout primes
For the analysis in Figure S4B, we established a set of kinematic primes (termed zero readout primes) from which no information could be read out. These primes were selected with the stringent double criterion that behavioural discrimination performance in the 2AFC intention discrimination task was at chance level ($p > 0.05$) across trials, and that intention readout values at the single-video, single subject level did not exceed the 95th percentile of the null-hypothesis distribution. Specifically, the significance of information readout for a given prime was established by creating a null-hypothesis distribution running 10000 times a logistic readout model on surrogate data in which intention choices were the same as in the original data for all primes but the one considered. For this prime, intention choices were generated at random, with equal probability between intention choices. This procedure ensures that readout is identical for actual data and surrogate data, with the only exception of the considered prime for which readout is erased by choice randomization.

Assessment of statistical differences

Data exclusions. Primed action categorization task
Trials in which the action was not correctly categorized were removed (2%). For the analysis of RTs, responses exceeding > 2.5 SD from the participant's mean were excluded from subsequent analysis (2%). For the analysis of initial fixations, trials in which no initial fixation on the probe was detected were excluded from the analysis (14%).

Gamma Mixed Effects Models (GMEM) for assessing statistical differences in RTs in the primed action categorization task
We used gamma mixed effects models to assess the significance of differences in RTs in the primed action discrimination task (Figure 1D; Tables S1–S3). Gamma distributions model positively skewed, non-negative data.[47] We compared gamma distributions with other distributions which allow for skewness (e.g., inverse gaussian, lognormal, etc.) and verified that models with gamma distributions yielded better performed both in terms of log-likelihood and BIC. To characterize the expected relationship between the predictors and the dependent variable, following the recommendations of Lo and Andrews,[48] we used an identity link function assuming RTs to be linearly affected by the predictors in the model (as opposed to non-linear alternatives such a log or inverse links). GMEM were implemented using the *glmer* function from the R package "lme4"[49] (https://cran.r-project.org/web/packages/lme4/index.html, 1.1-27.1).

To investigate the presence of kinematic priming at the single-participant, single-trial level in the primed action categorization task, we considered RT in each trial as dependent variable, kinematic prime and action probe as categorical predictors, as well as a prime by probe product term, and participant and prime identity as random effects (see Mixed Effects Model Random effects for further details). Categorical factors were specified using sum contrasts. In linear models for factors with two levels, sum contrasts provide a simple test for the difference between those two factor levels.[50]

We also used GMEM to quantify the dependence of RTs in the primed action categorization task on the single-trial kinematic encoding (readout) and the kinematic prime/action probe congruency (Figures 3A and 3B; Tables S1–S3). We considered RTs as dependent variable, action probe (drink, pour) and congruency (congruent, incongruent) as categorical predictors, and single-trial encoding (readout) as continuous predictor. To test the hypothesis of graded influence of single-trial intention encoding (readout) on congruency, all two and three-way interaction product terms were also added as fixed effects.

To quantify the influence of pre-video fluctuations in baseline pupil dilation, we extended the above model of RT (dependent variable) to include baseline pupil dilation (z-scored within each participant), in addition to single-trial readout as continuous predictors and congruency (congruent, incongruent) as a categorical predictor. To examine if there was any influence between pupil dilation, congruency and readout, all two and three-way interaction product terms were also included as fixed effects (Tables S1–S3).

We used GMEM to compare the amount of priming (quantified as differences in RTs between incongruent and congruent trials) between trials with single-trial encoding (readout) higher than the median value of single-trial encoding (readout) computed across all trials and participants and trials single-trial readout lower than this median value (Figures 3C and 3D; Tables S1–S3). Action probe was included as predictor to account for any potential biases it may induce.

Finally, we used GMEM to examine the priming effect in null readout primes using congruency (congruent, incongruent) and zero readout (zero as determined in *Null readout primes*; high as above with median split) as categorical predictors and RT in each trial as dependent variable (Figure S4B; Tables S1–S3).

Logistic Mixed Effects Models (LMEM) for assessing statistical differences in the distribution of binary variables

For assessing the significance of differences in initial fixations (Figures 4B, 4C, and 5A–5D; Tables S1–S3), we used logistic models. Logistic statistics were used because these quantities are computed from binary stochastic variables in each trial and thus cannot be assessed with t-tests or other parametric Gaussian statistics. For binary logistic regression, we fit our mixed models using a binomial distribution with a logit link function. Firstly, we considered fixation in the quadrant relevant for the displayed probe. A value of 1 was assigned if the initial fixation landed on the task-relevant quadrant of the action probe image displayed in that trial, and a value of 0 was assigned if the fixation landed in any of the other 3 quadrants (Figure 4A). Secondly, we considered fixation in the quadrant relevant for the not displayed probe. A value of 1 was assigned if the initial fixation landed on the task-relevant quadrant of the action probe image not displayed in that trial, and a value of 0 was assigned if the fixation landed in any of the other 3 quadrants (Figure 4A).

We also used LMEM to assess the significance of trial-level intention discrimination performance (Figure 1E), kinematic discrimination performance (Figure S1B) and readout model performance (Figures 2B and S2D; Tables S1–S3; encoding performance was not statistically tested as the model had perfect accuracy). For intention discrimination performance we considered single-trial correct choice (0/1) as the dependent variable and kinematic prime (drinking, pouring) as a categorical predictor. For readout model performance we considered single-trial correct prediction (0/1) as the dependent variable and kinematic prime (drinking, pouring) as a categorical predictor. For kinematic discrimination performance we considered single-trial correct choice (0/1) as the dependent variable but included no fixed effects, only participant specific random intercepts.

LMEM were implemented using the *glmer* function from the R package “lme4”[49] (https://cran.r-project.org/web/packages/lme4/index.html, 1.1-27.1).

Cumulative Link Mixed Model (CLMM) for assessing statistical differences in the distribution of ordinal data

Confidence rating by participants in the intention discrimination task are ordinal data. To examine the dependence of confidence rating on single-intention encoding (readout) (Figures 2F and S2E), we fit a CLMM with a logit link function, using the function *clmm* from the R package “ordinal” (https://CRAN.R-project.org/package=ordinal, 2019.12-10). We used the model to estimate the probability of each confidence rating for different values of single-trial intention encoding (readout). Prior to fitting the model, we collapsed confidence ratings 1 and 2, as these levels had much fewer responses. This was expected based on the relative high performance in the intention discrimination task (Figure 1E).

Mixed Model Fixed Effects

The significance of all fixed effects was assessed by conducting likelihood-ratio tests (LRT) between mixed models differing only in the presence or absence of the given predictor. Interactions were examined using the R package “emmeans” (https://CRAN.R-project.org/package=emmeans, 1.7.2), which provides post-hoc estimates of slopes for interactions in linear models and estimates of marginal means for predicted probabilities in logistic models. In logistic models the magnitude of the effect associated with a specific explanatory variable is not a constant value on the probability scale and the significance of product term coefficients does not guarantee the significance of interaction effects.[51–53] We thus quantified the size of the effects of interest by computing the predicted probabilities from the LMEMs of each independent variable in the interaction across all values of the other. We then computed the significance of the interaction effects on the probability scale by testing the equality of the marginal effects using a Wald test.

Mixed Model Random Effects

To determine the random-effects structure best supported by the data, we began with the minimum model that would account for non-independence of measurements, intercept only random effects for participant and kinematic prime (1|participant+1|prime), and incrementally added predictors. For establishing kinematic priming, we considered random participant slopes for action probe intention and kinematic prime intention and random prime slopes for action probe intention. We evaluated the model using all possible combinations of intercepts only/slopes and then ranked the model performance based on BIC. As final model, we selected the model with the lowest BIC that achieved convergence and for which all lower order models (with single term deletions) also converged so that we could use LRT to assess the significance of the effects. To examine the effect of encoding (readout) of intention information on kinematic priming, we included single-trial intention encoding (readout) for trends or encoding (readout) level (for categorical), along with congruency and probe intention, as candidate slopes for participants and probe intention as potential slope for the prime random effect. The final model used in each case is described in Table S2.

Significance of correlations

The significance of correlation values was assessed using the R *cor.test* function, with two-sided parametric Student’s *t* statistics for Pearson correlation and the asymptotic *t* approximation for Spearman correlation.

Conventions for p values

The p values of all reported statistical comparisons are two-sided, and Holm-Bonferroni corrected. Tables S1–S3 report the details of the Mixed Effects Models: Table S1 provides a summary of likelihood ratio tests for significance of main effects and product terms, Table S2 provides a summary of model formula and fixed effect coefficients, and Table S3 provides a summary of post-hoc tests of main effects/trends and interactions. In all Figures, * indicates $p < 0.05$, ** indicates $p < 0.01$, *** indicates $p < 0.001$.

Current Biology, Volume *33*

Supplemental Information

Kinematic priming of action predictions

Eugenio Scaliti, Kiri Pullar, Giulia Borghini, Andrea Cavallo, Stefano Panzeri, and Cristina Becchio

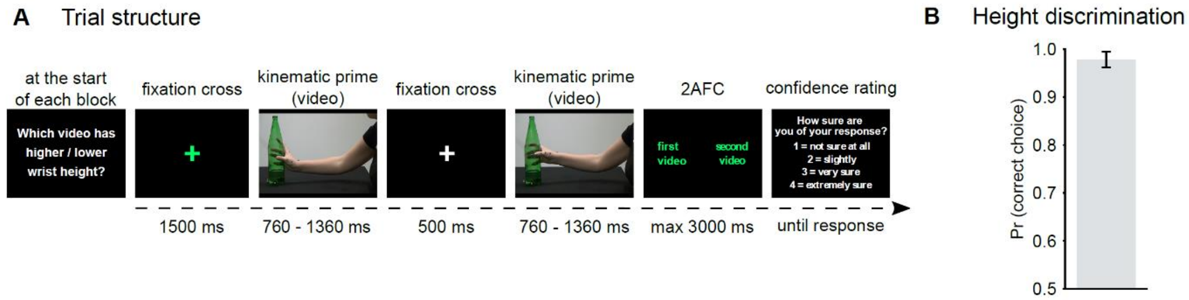

Figure S1. Wrist height discrimination task, Related to STAR Methods. Wrist height was the most informative kinematic variable and also the most readout variable. To verify that differences in wrist height were accessible to human perceivers, in a control study, we asked participants (N = 8) to discriminate wrist height in a 2AFC task. **A)** Trial structure. On each trial, participants observed two reach-to-grasp movements in two consecutive intervals. One interval contained a reach-to-pour, the other a reach-to-drink (in randomized order). Participants were asked to indicate the interval displaying the reach-to-grasp with higher peak wrist height. **B)** Wrist height discrimination performance quantified as the predicted probability of correct choice. The histogram shows the estimated marginal mean ± SE at the population-level estimated from a mixed model fit to single trial data.

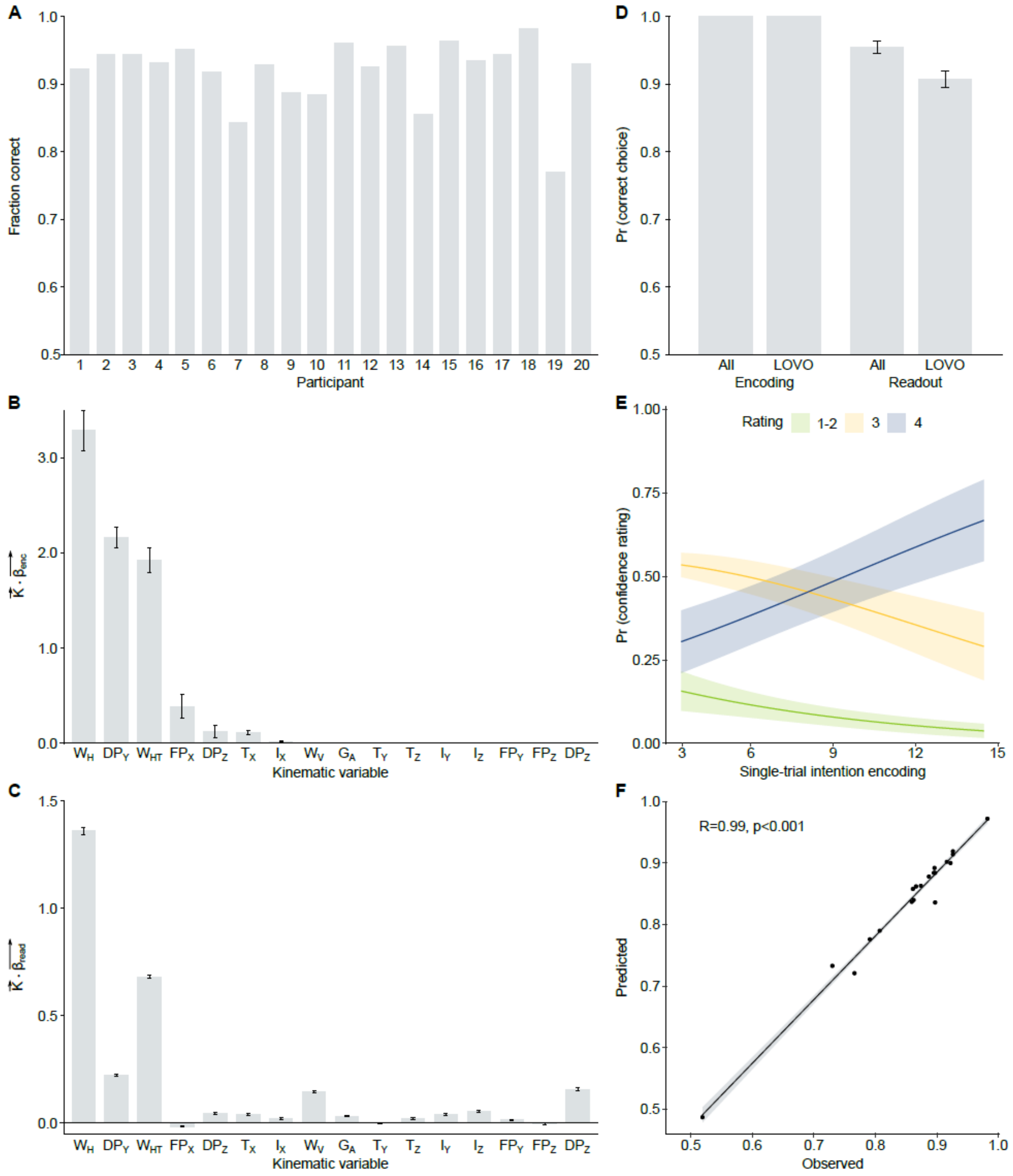

Figure S2. Kinematic intersection framework, Related to Figure 2. Here we summarize results of additional analyses to validate the modelling of intention encoding and intention readout using the kinematic intersection framework. **A)** Performance of the individual readout models trained on each participant. **B)** Contribution of individual kinematic variables to encoding computed as the scalar product between the encoding vector and the single-trial kinematic vector. Histograms represent mean ± SEM across kinematic primes. **C)** Contribution of individual kinematic variables to readout computed as the scalar product between the readout vector and the single-trial kinematic vector. Histograms represent mean ± SEM across trials and participants. **D)** Encoding (left) and readout (right) performance of models refit on entire data set after selection of λ parameter (ALL, as reported in main text) versus encoding/readout performance obtained using nested leave-one-video-out cross validation (LOVO). **E)** Probability of confidence rating in the intention discrimination task as a function of single-trial intention encoding. **F)** Pearson correlation between the observed and predicted (by the readout models) intention discrimination accuracies of individual perceivers. In Panels **E)**–**F)**, the line and shaded region correspond to estimated marginal mean ± SE estimated from the cumulative link mixed model and the linear model fit, respectively.

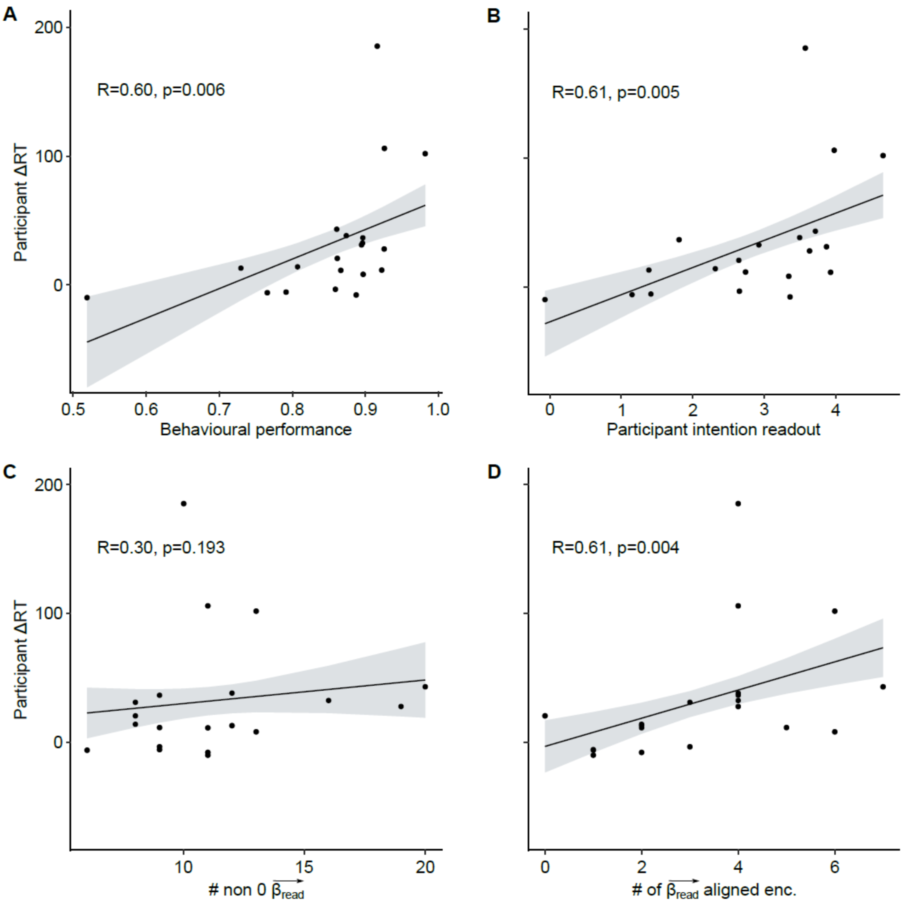

Figure S3. Intention readout predicts differences in kinematic priming across perceivers, Related to Figure 3. Here we summarize the results of several analyses that further validate the power of individual readout models trained on data from the intention discrimination task to predict RTs of the same perceiver in the primed action categorization task. **A)** Spearman correlation between individual intention discrimination performance and individual kinematic priming effect (RTs incongruent – RTs congruent). **B)** Spearman correlation between participant intention readout and individual kinematic priming effect. **C)** Spearman correlation between the number of non-zero readout coefficients and individual kinematic priming effect. **D)** Spearman correlation between the number of non-zero readout coefficients assigned to informative features and correctly aligned with encoding and individual kinematic priming effect. In all panels, the lines and shaded regions correspond to estimated marginal means ± SE estimated from linear model fit.

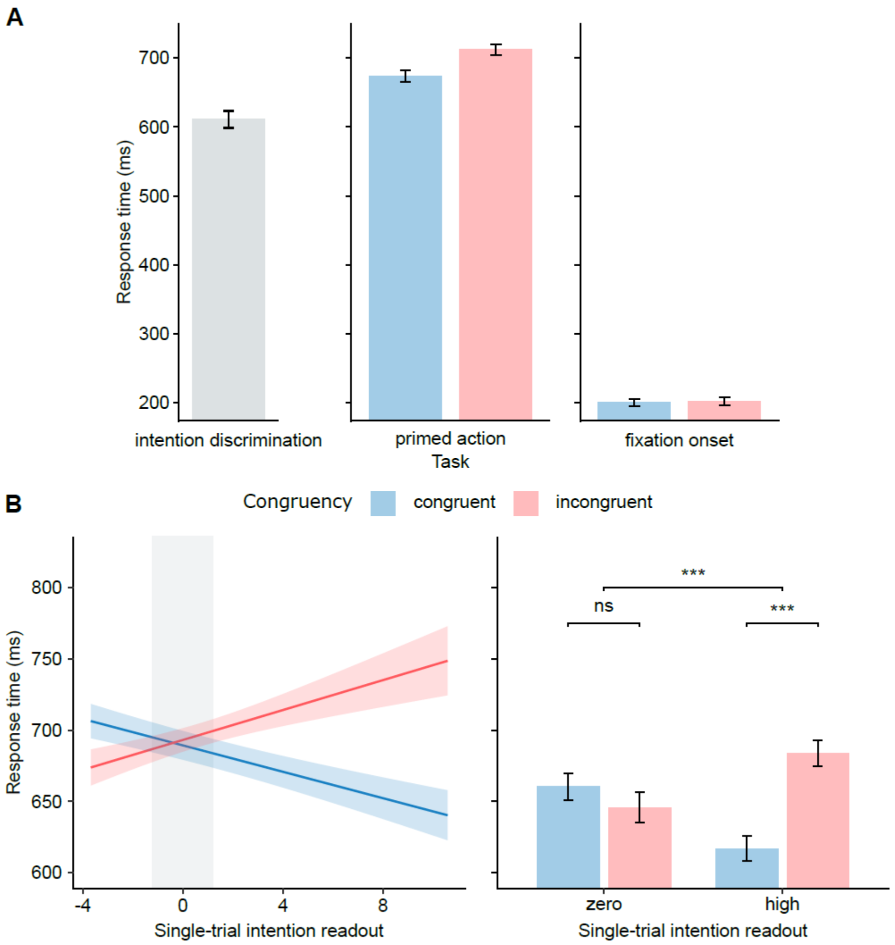

Figure S4. Additional analyses on response latencies, Related to Figures 3 and 4. Here we summarize the results of various analyses comparing response latencies across tasks and conditions. **A)** Left: RTs in the intention discrimination task. Middle: RTs in the primed action categorization task, separately for congruent and incongruent trials. Right: onset of initial fixation in the primed action categorization task, separately for congruent and incongruent trials. Initial fixations landed on the region predicted to contain task-relevant information approximately 200 ms after the probe was displayed. Because the time to discriminate kinematic primes in the intention discrimination task exceeded 600 ms, this observation refutes the idea that participants performed the primed action categorization task by first explicitly identifying the kinematic prime intention and then using this information to categorize the probe image intention. **B)** Left: RTs of the primed action categorization task plotted by single-trial intention readout and congruency. These data are the same as those in Fig 3B but are replotted here with the addition of the region (grey area) of single-trial intention readout values selected as zero-readout region for the selection of zero readout video primes. Right: RTs for the primed action categorization task plotted by congruency, separately for zero and high readout information. In comparison to zero-readout trials, RTs were faster on congruent trials and slower on incongruent trials in high-readout trials. This indicates that intention information read out in kinematic primes facilitated the processing of congruent probes and hindered the processing of incongruent probes.

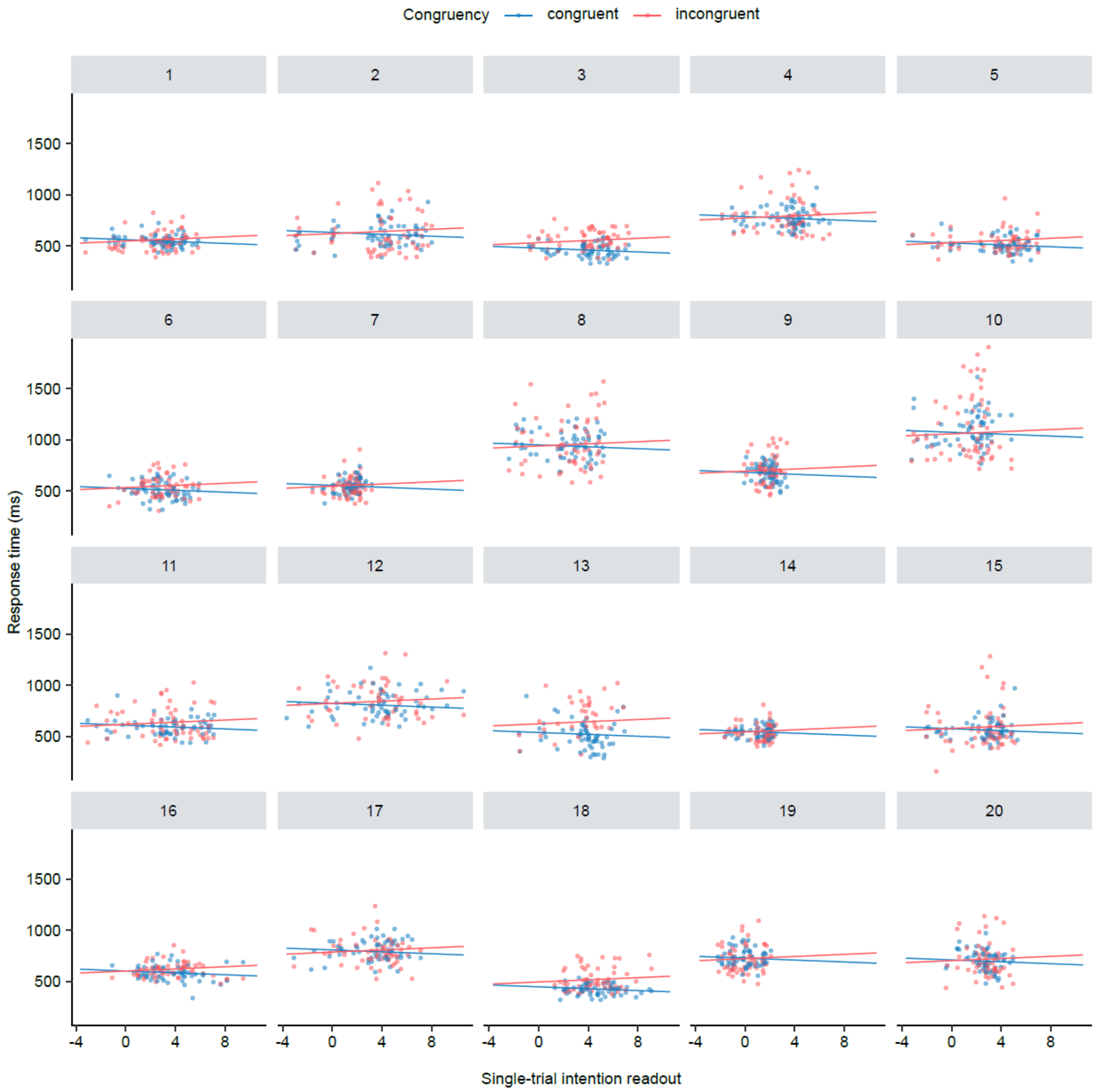

Figure S5. RT data for the primed action categorization task, Related to Figure 3. The figure displays the RT data for the primed action categorization task and demonstrates the consistency of the single-trial intention readout priming effect across individual perceivers (identified by grey boxes). Each panel depicts RTs by single-trial intention readout and congruency for each perceiver, with individual trials represented as data points. The red and blue lines plot the conditional marginal means estimated from the mixed model fit to single trial data for congruent and incongruent trials, respectively. The trend reported at the population level in Figure 3B is highly consistent across individual perceivers.

RTs: dependence on prime and probe (Fig. 1D)

	χ^2	df	p
**Probe**	**4.534**	**1**	**0.033**
Prime	1.493	1	0.222
**Probe:Prime**	**64.896**	**1**	**<0.001**

Intention discrimination accuracy (Fig. 1E)

	χ^2	df	p
**Prime**	**10.693**	**1**	**0.001**

Confidence ratings: dependence on single-trial intention encoding and readout (Fig. S2E and 2F)

	χ^2	df	p
**Encoding**	**4.839**	**1**	**0.0278**

	χ^2	df	p
**Readout**	**37.022**	**1**	**<0.001**

RTs: dependence on congruency and single-trial intention encoding and readout (Fig. 3A and 3B)

	χ^2	df	p
Encoding	0.060	1	0.807
Congr	3.441	1	0.064
Probe	0.333	1	0.564
Encoding:Congr	0.367	1	0.545
**Encoding:Probe**	**5.772**	**1**	**0.016**
Congr:Probe	0.336	1	0.562
Encoding:Congr:Probe	0.046	1	0.831

	χ^2	df	p
Readout	0.037	1	0.847
Congr	0.062	1	0.803
**Probe**	**4.405**	**1**	**0.036**
**Readout:Congr**	**12.749**	**1**	**<0.001**
Readout:Probe	0.526	1	0.468
Congr:Probe	0.877	1	0.349
Readout:Congr:Probe	0.109	1	0.741

RT Priming effect: comparison between low and high single-trial intention encoding and readout (Fig. 3C and 3D)

	χ^2	df	p
EncLev	0.188	1	0.664
**Congr**	**59.279**	**1**	**<0.001**
**Probe**	**4.386**	**1**	**0.036**
EncLev:Congr	0.439	1	0.508
**EncLev:Probe**	**4.328**	**1**	**0.037**
Congr:Probe	1.354	1	0.245
EncLev:Congr:Probe	0.576	1	0.448

	χ^2	df	p
ReadLev	0.004	1	0.950
**Congr**	**59.221**	**1**	**<0.001**
**Probe**	**24.491**	**1**	**<0.001**
**ReadLev:Congr**	**36.475**	**1**	**<0.001**
ReadLev:Probe	1.018	1	0.313
Congr:Probe	1.344	1	0.246
ReadLev:Congr:Probe	0.070	1	0.791

Fixation in quadrant relevant for displayed probe: dependence on prime and probe (logit scale; Fig. 4B)

	χ^2	df	p
Probe	0.445	1	0.505
Prime	0.569	1	0.451
**Probe:Prime**	**60.961**	**1**	**<0.001**

Fixation in quadrant relevant for not displayed probe: dependence on prime and probe (logit scale; Fig. 4C)

	χ^2	df	p
Probe	1.123	1	0.289
Prime	1.347	1	0.246
**Probe:Prime**	**53.190**	**1**	**<0.001**

Initial fixations: dependence on single-trial intention encoding and readout (logit scale; Fig. 5A and 5B)

	χ^2	df	p
Encoding	0.832	1	0.362
Congr	0.812	1	0.368
Probe	1.433	1	0.231
Encoding:Congr	2.379	1	0.123
Encoding:Probe	1.018	1	0.313
Congr:Probe	2.944	1	0.086
**Encoding:Congr:Probe**	**4.448**	**1**	**0.035**

	χ^2	df	p
Readout	3.669	1	0.055
Congr	2.709	1	0.100
Probe	0.100	1	0.751
**Readout:Congr**	**11.445**	**1**	**<0.001**
Readout:Probe	0.004	1	0.947
Congr:Probe	1.545	1	0.214
Readout:Congr:Probe	3.171	1	0.075

Initial fixations priming effect: comparison between low and high intention encoding and readout (logit scale; Fig. 5C and 5D)

	χ^2	df	p
EncLev	1.010	1	0.315
**Congr**	**58.021**	**1**	**<0.001**
Probe	0.514	1	0.474
EncLev:Congr	2.448	1	0.118
EncLev:Probe	0.945	1	0.331
Congr:Probe	0.824	1	0.364
EncLev:Congr:Probe	1.052	1	0.305

	χ^2	df	p
ReadLev	0.394	1	0.530
**Congr**	**60.179**	**1**	**<0.001**
Probe	0.213	1	0.644
**ReadLev:Congr**	**13.031**	**1**	**<0.001**
ReadLev:Probe	0.604	1	0.437
Congr:Probe	0.330	1	0.566
ReadLev:Congr:Probe	0.061	1	0.805

RT Priming effect: comparison between zero and high single-trial intention readout (Fig. S4B)

	χ^2	df	p
ZeroRead	0.068	1	0.794
**Congr**	**13.103**	**1**	**<0.001**
**ZeroRead:Congr**	**27.620**	**1**	**<0.001**

Pooled participants: RTs dependence on congruency and single-trial intention readout (not plotted in Figures)

	χ^2	df	p
Readout	0.143	1	0.706

Pupil dilation: RTs dependence on congruency and single-trial intention readout (not plotted in Figures)

	χ^2	df	p
Readout	1.012	1	0.314

**Congr**	**8.119**	**1**	**0.004**
Probe	0.002	1	0.969
Readout:Congr	0.734	1	0.392
**Readout:Probe**	**6.675**	**1**	**0.010**
Congr:Probe	0.102	1	0.750
Readout:Congr:Probe	0.174	1	0.677

Congr	0.085	1	0.770
**Pupil**	**14.799**	**1**	**<0.001**
**Reaout:Congr**	**26.107**	**1**	**<0.001**
Readout:Pupil	0.134	1	0.714
Congr:Pupil	2.539	1	0.111
Readout:Congr:Pupil	0.004	1	0.951

Table S1. Summary of likelihood ratio tests for significance of main effects and product terms in mixed effects statistics, Related to Figures 1-5.

RTs: dependence on prime and probe (Fig. 1D)

RT~Probe*Prime+(1+Probe|participant)+(1|prime)

	est	SE	z	p
**(Intercept)**	**692.843**	**7.607**	**91.077**	**<0.001**
**Probe1**	**-12.593**	**4.558**	**-2.763**	**0.006**
Prime1	-4.483	3.617	-1.240	0.215
**Probe1:Prime1**	**-18.984**	**2.331**	**-8.144**	**<0.001**

Intention discrimination accuracy (Fig.1E)

Accuracy~Prime+(1+Prime|participant)+(1|prime)

	est	SE	z	p
**(Intercept)**	**2.804**	**0.284**	**9.870**	**<0.001**
**Prime1**	**-0.899**	**0.261**	**-3.440**	**<0.001**

Confidence ratings: dependence on single-trial intention encoding and readout (Fig. S2E and 2F)

ConfRating~Encoding+(1+Encoding|participant)+(1|prime)

	est	SE	z	p
**Encoding**	**0.138**	**0.062**	**2.235**	**0.025**
Threshold coeff 1-2|3	-1.191	0.593	-2.008	
Threshold coeff 3|4	1.298	0.593	2.188	

ConfRating~Readout+(1+Readout|participant)+(Readout|prime)

	est	SE	z	p
**Readout**	**0.700**	**0.076**	**9.221**	**<0.001**
Threshold coeff 1-2|3	-0.414	0.375	-1.104	
Threshold coeff 3|4	2.372	0.378	6.278	

RTs: dependence on congruency and single-trial intention encoding and readout (Fig. 3A and 3B)

RT~Encoding*Congr*Probe+(1+Probe|participant)+(1|prime)

	est	SE	z	p
**(Intercept)**	**689.894**	**9.443**	**73.057**	**<0.001**
Encoding	0.347	1.344	0.258	0.797
**Congr1**	**-13.424**	**5.992**	**-2.240**	**0.025**
Probe1	5.603	6.253	0.896	0.370
Encoding:Congr1	-0.567	0.744	-0.763	0.445
**Encoding:Probe1**	**-2.257**	**0.756**	**-2.984**	**0.003**
Congr1:Probe1	-7.021	7.960	-0.882	0.378
Encoding:Congr1:Probe1	0.303	1.007	0.301	0.763

RT~Readout*Congr*Probe+(1+Congr|participant)+(1|prime)

	est	SE	z	p
**(Intercept)**	**691.206**	**7.533**	**91.761**	**<0.001**
Readout	0.308	1.495	0.206	0.837
Congr1	-1.884	5.280	-0.357	0.721
**Probe1**	**-9.795**	**3.508**	**-2.792**	**0.005**
**Readout:Congr1**	**-4.942**	**1.203**	**-4.108**	**<0.001**
Readout:Probe1	-0.913	1.039	-0.879	0.379
Congr1:Probe1	-5.295	4.356	-1.215	0.224
Readout:Congr1:Probe1	0.461	1.196	0.386	0.700

RT Priming effect: comparison between low and high single-trial intention encoding and readout (Fig. 3C and 3D)

RT~EncLev*Congr*Probe+(1+Probe|participant)+(1|prime)

	est	SE	z	p
**(Intercept)**	**692.446**	**7.288**	**95.015**	**<0.001**
EncLev1	1.585	3.081	0.514	0.607
**Congr1**	**-18.272**	**2.301**	**-7.942**	**<0.001**
**Probe1**	**-12.358**	**3.972**	**-3.111**	**0.002**
EncLev1:Congr1	1.599	2.251	0.710	0.477
**EncLev1:Probe1**	**5.034**	**2.254**	**2.233**	**0.026**
Congr1:Probe1	-4.268	3.206	-1.331	0.183
EncLev1:Congr1:Probe1	-2.780	3.370	-0.825	0.409

RT~ReadLev*Congr*Probe+(1+Congr|participant)+(1|prime)

	est	SE	z	p
**(Intercept)**	**691.182**	**5.768**	**119.822**	**<0.001**
ReadLev1	0.213	2.836	0.075	0.940
**Congr1**	**-18.606**	**2.345**	**-7.933**	**<0.001**
**Probe1**	**-12.219**	**2.232**	**-5.474**	**<0.001**
**ReadLev1:Congr1**	**14.922**	**2.217**	**6.730**	**<0.001**
ReadLev1:Probe1	2.485	2.306	1.077	0.281
Congr1:Probe1	-4.242	3.322	-1.277	0.202
ReadLev1:Congr1:Probe1	-0.716	2.450	-0.292	0.770

Fixation in quadrant relevant for displayed probe: dependence on prime and probe (Fig. 4B)

Fixation~Probe*Prime+(1+Probe|participant)+(1|prime)

	est	SE	z	p
**(Intercept)**	**0.644**	**0.211**	**3.050**	**0.002**
Probe1	-0.119	0.177	-0.671	0.502
Prime1	-0.042	0.055	-0.758	0.448
**Probe1:Prime1**	**0.340**	**0.043**	**7.854**	**<0.001**

Fixation in quadrant relevant for not displayed probe: dependence on prime and probe (Fig. 4C)

Fixation~Probe*Prime+(1+Probe|participant)+(1|prime)

	est	SE	z	p
**(Intercept)**	**-2.598**	**0.145**	**-17.891**	**<0.001**
Probe1	-0.239	0.222	-1.079	0.281
Prime1	-0.092	0.078	-1.182	0.237
**Probe1:Prime1**	**-0.472**	**0.062**	**-7.586**	**<0.001**

Initial fixations: dependence on single-trial intention encoding and readout (logit scale; Fig. 5A and 5B)

Fixation~Encoding*Congr*Probe+(1+Probe|participant)+(1|prime)

	est	SE	z	p
(Intercept)	0.516	0.267	1.932	0.053
Encoding	0.019	0.021	0.917	0.359
Congr1	0.127	0.140	0.906	0.365
Probe1	-0.268	0.222	-1.207	0.227
Encoding:Congr1	0.026	0.017	1.550	0.121
Encoding:Probe1	0.017	0.017	1.013	0.311
Congr1:Probe1	0.299	0.172	1.732	0.083
**Encoding:Congr1:Probe1**	**-0.044**	**0.021**	**-2.138**	**0.032**

Fixation~Readout*Congr*Probe+(1+Probe|participant)+(1|prime)

	est	SE	z	p
**(Intercept)**	**0.471**	**0.227**	**2.072**	**0.038**
Readout	0.052	0.027	1.926	0.054
Congr1	0.132	0.080	1.657	0.098
Probe1	-0.059	0.187	-0.317	0.751
**Readout:Congr1**	**0.075**	**0.022**	**3.387**	**<0.001**
Readout:Probe1	-0.002	0.024	-0.066	0.947
Congr1:Probe1	0.112	0.090	1.249	0.212
Readout:Congr1:Probe1	-0.042	0.024	-1.789	0.074

Initial fixations priming effect: comparison between low and high intention encoding and readout (logit scale; Fig. 5C and 5D)

Fixation~EncLev*Congr*Probe+(1+Probe|participant)+(1|prime)

	est	SE	z	p
**(Intercept)**	**0.652**	**0.211**	**3.088**	**0.002**
EncLev1	-0.055	0.054	-1.010	0.312
**Congr1**	**0.335**	**0.044**	**7.669**	**<0.001**
Probe1	-0.128	0.118	-0.721	0.471
EncLev1:Congr1	-0.068	0.043	-1.571	0.116
EncLev1:Probe1	-0.042	0.043	-0.976	0.329
Congr1:Probe1	-0.050	0.055	-0.913	0.361
EncLev1:Congr1:Probe1	0.056	0.054	1.032	0.302

Fixation~ReadLev*Congr*Probe+(1+Probe|participant)+(1|prime)

	est	SE	z	p
**(Intercept)**	**0.643**	**0.214**	**3.008**	**0.003**
ReadLev1	-0.036	0.056	-0.631	0.528
**Congr1**	**0.352**	**0.045**	**7.815**	**<0.001**
Probe1	-0.083	0.178	-0.464	0.643
**ReadLev1:Congr1**	**-0.165**	**0.046**	**-3.619**	**<0.001**
ReadLev1:Probe1	-0.041	0.053	-0.781	0.435
Congr1:Probe1	-0.032	0.056	-0.576	0.564
ReadLev1:Congr1:Probe1	-0.012	0.048	-0.248	0.804

Wrist height discrimination accuracy (Fig. S1B)

Accuracy~1+(1|participant)

	est	SE	z	p
**(Intercept)**	**3.812**	**0.776**	**4.909**	**<0.001**

RT Priming effect: comparison between zero and high single-trial intention readout (Fig. S4B)

RT~ZeroRead*Congr*Probe+(1+Probe|participant)+(1|prime)

	est	SE	z	p
**(Intercept)**	**651.604**	**6.958**	**93.644**	**<0.001**
ZeroRead1	1.349	4.428	0.305	0.761
**Congr1**	**-13.064**	**3.286**	**-3.976**	**<0.001**
**ZeroRead1:Congr1**	**20.427**	**3.490**	**5.853**	**<0.001**

Pooled participants: RTs dependence on congruency and single-trial intention readout (not plotted in Figures)

RT~Readout*Congr*Probe+(1+Probe|participant)+(1|prime)

	est	SE	z	p
**(Intercept)**	**694.143**	**6.072**	**114.326**	**<0.001**
Readout	-1.238	2.587	-0.478	0.632
**Congr1**	**-18.352**	**4.153**	**-4.419**	**<0.001**
Probe1	-0.322	4.127	-0.078	0.938
Readout:Congr1	-2.247	1.852	-1.214	0.225
**Readout:Probe1**	**-6.853**	**1.790**	**-3.828**	**<0.001**
Congr1:Probe1	-2.550	4.532	-0.563	0.574
Readout:Congr1:Probe1	-1.336	2.085	-0.641	0.522

Pupil dilation: RTs dependence on congruency and single-trial intention readout (not plotted in Figures)

RT~Readout*Congr*Pupil+(1|participant)+(1+Probe|prime)

	est	SE	z	p
**(Intercept)**	**668.453**	**7.264**	**92.017**	**<0.001**
Readout	1.406	1.319	1.066	0.286
Congr1	-1.611	4.143	-0.389	0.697
**Pupil**	**14.058**	**3.206**	**4.385**	**<0.001**
**Readout:Congr1**	**-6.402**	**1.150**	**-5.565**	**<0.001**
Readout:Pupil	-0.362	0.902	-0.401	0.688
Congr1:Pupil	5.656	3.283	1.722	0.085
Readout:Congr1:Pupil	0.061	0.925	0.066	0.948

Table S2. Summary of model formula and fixed coefficients for mixed effects statistics, Related to Figures 1-5.

RTs: effects of prime (Fig. 1D)

probe	est	SE	z	p
**drinking**	**-46.935**	**8.630**	**-5.439**	**<0.001**
**pouring**	**29.002**	**8.581**	**3.380**	**<0.001**

RTs: difference in priming effect (Fig. 1D)

	est	SE	z	p
**drinking -pouring**	**-75.937**	**9.324**	**-8.144**	**<0.001**

Intention discrimination (prob. scale; Fig. 1E)

	est	SE	z	p
**vs. chance (0.5)**	**0.943**	**0.015**	**28.958**	**<0.001**

RTs: effects of single-trial intention encoding and readout (Fig. 3A and 3B)

	Encoding trend	SE	z	p
congruent	-0.221	1.312	-0.168	1.000
incongruent	0.914	1.731	0.528	1.000

	Readout trend	SE	z	p
**congruent**	**-4.634**	**1.471**	**-3.151**	**0.003**
**incongruent**	**5.250**	**2.281**	**2.302**	**0.021**

RTs: effects of congruency (Fig. 3A and 3B)

	est	SE	z	p
incongruent - congruent	1.135	1.487	0.763	0.445

	est	SE	z	p
**incongruent - congruent**	**9.884**	**2.406**	**4.108**	**<0.001**

RT Priming effect: effects of low and high single-trial intention encoding and readout (Fig. 3C and 3D)

EncLev	est	SE	z	p
**low**	**33.345**	**6.446**	**5.173**	**<0.001**
**high**	**39.742**	**6.430**	**6.181**	**<0.001**

ReadLev	est	SE	z	p
low	7.369	6.476	1.138	0.255
**high**	**67.056**	**6.434**	**10.422**	**<0.001**

RT Priming effect: low vs. high single-trial intention encoding and readout (Fig. 3C and 3D)

	est	SE	z	p
low - high	-6.397	9.005	-0.710	0.477

	est	SE	z	p
**low - high**	**-59.686**	**8.869**	**-6.730**	**<0.001**

Fixation in quadrant relevant for displayed probe: effect of prime (prob. scale; Fig. 4B)

probe	est	SE	z	p
**drinking**	**0.138**	**0.035**	**4.002**	**<0.001**
**pouring**	**-0.164**	**0.034**	**-4.784**	**<0.001**

Fixation in quadrant relevant for displayed probe: difference in priming effect (prob. scale; Fig. 4B)

	est	SE	z	p
**drinking -pouring**	**0.303**	**0.044**	**6.827**	**<0.001**

Fixation in quadrant relevant for not displayed probe: effect of prime (prob. scale; Fig. 4C)

probe	est	SE	z	p
**drinking**	**-0.061**	**0.019**	**-3.281**	**0.002**
**pouring**	**0.061**	**0.021**	**2.872**	**0.004**

Fixation in quadrant relevant for not displayed probe: difference in priming effect (prob. scale; Fig. 4C)

	est	SE	z	p
**drinking -pouring**	**-0.122**	**0.024**	**-5.153**	**<0.001**

Initial fixation: effects of single-trial intention encoding and readout (prob. scale; Fig. 5A and 5B)

	Encoding trend	SE	z	p
congruent	0.008	0.004	1.934	0.106
incongruent	-0.002	0.007	-0.203	0.839

	Readout trend	SE	z	p
**congruent**	**0.025**	**0.007**	**3.822**	**<0.001**
incongruent	-0.006	0.010	-0.600	0.549

Initial fixations: effect of congruency (prob. scale; Fig. 5A and 5B)

	est	SE	z	p
incongruent - congruent	-0.010	0.008	-1.265	0.206

	est	SE	z	p
**incongruent - congruent**	**-0.031**	**0.011**	**-2.895**	**0.004**

Initial fixations: effects of low and high single-trial intention encoding and readout (prob. scale; Fig. 5C and 5D)

EncLev	est	SE	z	p
**low**	**-0.121**	**0.029**	**-4.138**	**<0.001**
**high**	**-0.175**	**0.031**	**-5.682**	**<0.001**

ReadLev	est	SE	z	p
**low**	**-0.084**	**0.030**	**-2.791**	**0.005**
**high**	**-0.227**	**0.030**	**-6.904**	**<0.001**

Initial fixations: low vs. high intention single trial intention encoding and readout (prob. scale; Fig. 5C and 5D)

	est	SE	z	p
low - high	0.054	0.041	1.331	0.183

	est	SE	z	p
**low - high**	**0.143**	**0.043**	**3.316**	**<0.001**

Wrist height discrimination (prob. scale; Fig. S1B)

	est	SE	z	p
**vs. chance (0.5)**	**0.978**	**0.0164**	**29.113**	**<.0001**

RT Priming effect: effects of zero and high single-trial intention readout (Fig. S4B)

	est	SE	z	p
zero	-14.726	11.759	-1.252	0.210
**high**	**66.982**	**6.749**	**9.925**	**<0.001**

RT readout information effect: effects of congruent and incongruent trials (Fig. S4B)

	est	SE	z	p
**congruent**	**-43.552**	**9.740**	**-4.471**	**<0.001**
**incongruent**	**38.156**	**12.625**	**3.022**	**0.005**

Pooled participants: RTs effects of single-trial intention readout (not plotted in Figures)

	Readout trend	SE	z	p
congruent	-3.485	2.664	-1.308	0.382
incongruent	1.010	3.625	0.279	0.781

Pupil dilation: RTs effects of single-trial intention readout (not plotted in Figures)

	Readout trend	SE	z	p
**congruent**	**-4.943**	**1.390**	**-3.555**	**<0.001**
**incongruent**	**7.882**	**2.034**	**3.876**	**<0.001**

Pooled participants: RTs effects of congruency (not plotted in Figures)

	est	SE	z	p
incongruent - congruent	4.495	3.704	1.214	0.225

Pupil dilation: RTs effects of congruency (not plotted in Figures)

	est	SE	z	p
**incongruent - congruent**	**12.825**	**2.292**	**5.596**	**<0.001**

Table S3. Summary of post-hoc tests of main effects/trends and interactions for mixed model analyses, Related to Figures 1-5.
